# *Drosophila* Clock Is Required in Brain Pacemaker Neurons to Prevent Premature Locomotor Aging Independently of Its Circadian Function

**DOI:** 10.1371/journal.pgen.1006507

**Published:** 2017-01-10

**Authors:** Alexandra Vaccaro, Abdul-Raouf Issa, Laurent Seugnet, Serge Birman, André Klarsfeld

**Affiliations:** 1 Genes Circuits Rhythms and Neuropathology, Brain Plasticity Unit, ESPCI Paris/CNRS, PSL Research University, Paris, France; 2 Integrated Physiology of the Brain Arousal Systems (WAKING), Lyon Neuroscience Research Centre, INSERM/CNRS/UCBL1, Lyon, France; Washington University in Saint Louis School of Medicine, UNITED STATES

## Abstract

Circadian clocks control many self-sustained rhythms in physiology and behavior with approximately 24-hour periodicity. In many organisms, oxidative stress and aging negatively impact the circadian system and sleep. Conversely, loss of the clock decreases resistance to oxidative stress, and may reduce lifespan and speed up brain aging and neurodegeneration. Here we examined the effects of clock disruptions on locomotor aging and longevity in *Drosophila*. We found that lifespan was similarly reduced in three arrhythmic mutants (*Clk*^AR^, *cyc*^0^ and *tim*^0^) and in wild-type flies under constant light, which stops the clock. In contrast, *Clk*^AR^ mutants showed significantly faster age-related locomotor deficits (as monitored by startle-induced climbing) than *cyc*^0^ and *tim*^0^, or than control flies under constant light. Reactive oxygen species accumulated more with age in *Clk*^AR^ mutant brains, but this did not appear to contribute to the accelerated locomotor decline of the mutant. Clk, but not Cyc, inactivation by RNA interference in the pigment-dispersing factor (PDF)-expressing central pacemaker neurons led to similar loss of climbing performance as *Clk*^AR^. Conversely, restoring Clk function in these cells was sufficient to rescue the *Clk*^AR^ locomotor phenotype, independently of behavioral rhythmicity. Accelerated locomotor decline of the *Clk*^AR^ mutant required expression of the PDF receptor and correlated to an apparent loss of dopaminergic neurons in the posterior protocerebral lateral 1 (PPL1) clusters. This neuronal loss was rescued when the *Clk*^AR^ mutation was placed in an apoptosis-deficient background. Impairing dopamine synthesis in a single pair of PPL1 neurons that innervate the mushroom bodies accelerated locomotor decline in otherwise wild-type flies. Our results therefore reveal a novel circadian-independent requirement for Clk in brain circadian neurons to maintain a subset of dopaminergic cells and avoid premature locomotor aging in *Drosophila*.

## Author Summary

Circadian clocks are highly conserved from flies to humans. They control rhythms in most physiological functions, with free-running periods close to 24 h. Clock disruption, as occurs in shift-work or jet lag, is increasingly suspected to reduce healthspan. Aging and neurodegenerative disorders, like Parkinson’s disease, often disrupt biological clocks early on. It is thus important to understand, and eventually block, a vicious circle that could contribute to accelerated aging. Here we studied the effects of mutations in three *Drosophila* core circadian genes (*timeless*, *cycle* and *Clock*) on lifespan and age-related locomotor impairment, an almost universal hallmark of animal and human senescence. We found that expression of *Clock* in the main circadian pacemaker neurons is necessary to avoid premature locomotor aging, independently of its circadian function. *Clock* deficiency in circadian neurons disrupted specific dopaminergic neurons in aging mutant flies, with the involvement of pro-apoptotic pathways, while inhibiting dopamine synthesis in a single pair of these neurons also led to early locomotor decline. This reveals an unexpected link between circadian and dopaminergic circuits in the fly brain, which may be of broad significance to unravel the reciprocal influence between two prominent pathological features of Parkinson's disease: circadian system dysfunction and dopaminergic neurodegeneration.

## Introduction

Circadian clocks are ubiquitous in the living world, driving rhythms at many levels, from the molecular to the behavioral [1]. The main defining characteristics of these clocks are that: (i) they are self-sustained, ticking for many days in constant external conditions, (ii) their period in such conditions, called free-run, is close to 24 h. Circadian clocks are normally synchronized to the solar day, ensuring appropriate timing of the processes they control. Light and temperature are the main "Zeitgebers", or synchronizing signals.

The cell-autonomous machineries of circadian clocks have been described in great detail in many taxa, including bacteria, plants, mammals and insects [2, 3]. They usually include negative transcriptional feedback loops, which are exceedingly well conserved evolutionarily. In *Drosophila*, one of these feedback loops involves a pair of transcriptional activation factors, Clock (Clk) and Cycle (Cyc). These basic-helix-loop-helix (bHLH) proteins form heterodimers via their PAS interaction domains (reviewed in [3]). Clk-Cyc dimers activate the *period* (*per*) and *timeless* (*tim*) genes, which are subsequently turned off by their own gene products, as these also form dimers that bind to and inhibit Clk-Cyc. Post-transcriptional mechanisms introduce appropriate delays to allow inhibition to start only after the *per* and *tim* mRNAs have accumulated to high levels. Both the latter mechanisms and the feedback loop itself are homologous in mammals, except for the replacement of *tim* by *cryptochromes* (and the naming of *cyc* as Bmal1).

Clocks may allow organisms both to better adjust to predictable nycthemeral changes in their environment, and to achieve some temporal order in their functioning, independently of solar cycles [4]. In any case, the functional importance of clocks is attested by the numerous adverse effects of perturbing them on healthspan, both in insects [4–10] and rodents [11–16]. This ever-increasing list is consistent with the presence in all tissues of functional clocks, controlling up to 10% of the genes expressed in any given tissue [17]. Experimental clock disruptions can be genetic, but also environmental, e.g. with constant light (LL) that stops the clocks by activating Tim degradation [1, 2], or with light-dark (LD) cycles of non-24h periodicity, or which are shifted by several hours every few days to generate chronic jet lag-like conditions.

In humans, epidemiological data, although only correlative, also suggest that chronic circadian disruption, such as in long-term shift work, increases the incidence of obesity (and metabolic syndrome more generally), cardiovascular diseases, and some cancers, to name only a few pathologies [18]. In large cities, artificial light at night, combined with weak indoor light during the day, may also sufficiently disrupt our circadian clocks to produce similar, if weaker, ill effects on the general population [15].

Age-related locomotor declines or impairments (ARLI), which have a strong impact on life quality, are found in most species. They are thus often used to assay functional aging, particularly in insects (reviewed in [19]). Dopaminergic (DA) circuits are important for proper motor control and abilities, both in mammals [20–22] and *Drosophila* [23–27]. This importance is illustrated by the devastating effect of specific DA neuronal loss on motor control in Parkinson's disease (PD), for which age is the major risk factor [28]. There are perturbations of the circadian system in neurodegenerative diseases, including PD (sometimes before any motor symptoms), and their animal models, as reviewed [29]. The inverse relationship–circadian disruption causing neuronal loss–remains hypothetical, but is consistent with a recent study in mice [30].

Here, we studied mutations in the two *Drosophila* circadian transcriptional activators Clk and Cyc, and in one of their target genes, *tim*. Male flies were kept either in standard LD cycles or in LL, and assayed at different ages for survival, locomotor performance, brain reactive oxygen species (ROS) levels, and size of brain dopaminergic neuronal subpopulations. Our results confirmed the negative impact of genetically- or environmentally-imposed arrhythmia on healthspan. They also revealed unexpected circadian rhythm- and Cyc-independent effects of *Clk* gene disruption, namely increased brain ROS levels and a markedly accelerated decline of locomotor responses in aging flies. The latter phenotype was attributed specifically to *Clk* function in the small lateral ventral neurons (s-LNv), which express the neuropeptide PDF and constitute an important circadian pacemaker in the *Drosophila* brain, and may be accounted for by the observed effect of *Clk* inactivation on the PPL1 clusters of brain dopaminergic neurons.

## Results

### The *Clk*^AR^ mutation has a stronger effect on ARLI than other clock disruptions

**Fig 1:**
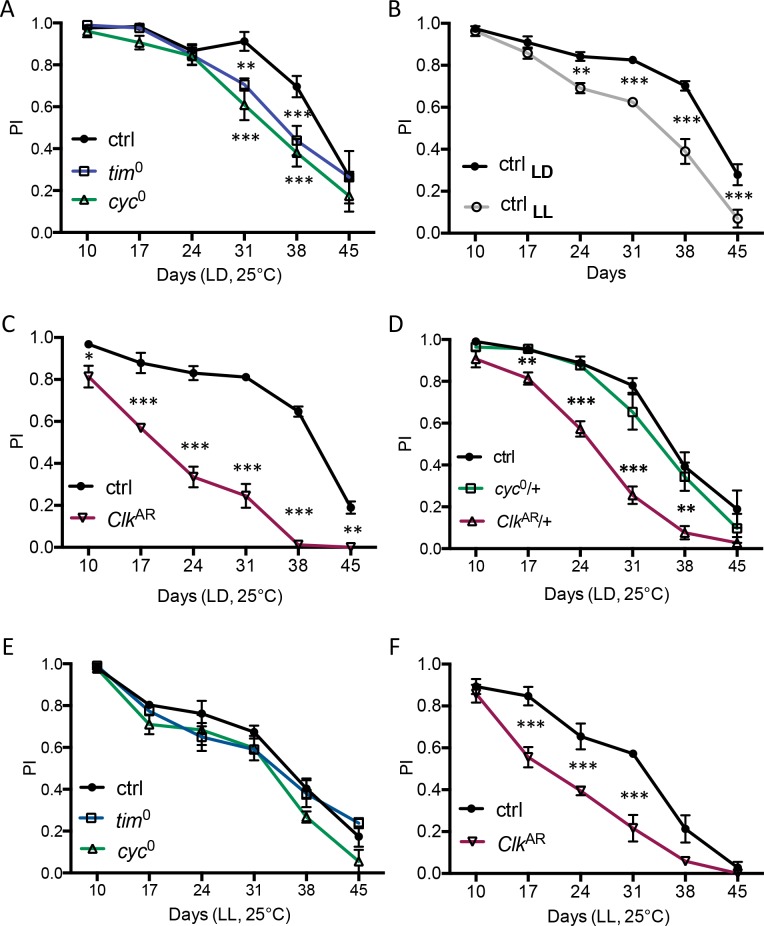
Age-related locomotor impairment (ARLI) in *cyc*^0^, *tim*^0^ and *Clk*^AR^ arrhythmic mutants. (A) ARLI of *cyc*^0^ and *tim*^0^ mutants is accelerated relative to wild-type flies (ctrl) housed in LD. (B) ARLI of wild-type Canton-S flies (ctrl) is accelerated under constant light (LL) that stops the clock. (C) The effect of *Clk*^AR^ mutation on ARLI is stronger and occurs earlier than the effect of *cyc*^0^, *tim*^0^ or LL. (D) *Clk*^AR^/+, but not *cyc*^0^/+, heterozygote mutants also exhibit an accelerated locomotor decline as compared to controls. (E) No effect on ARLI is observed for *cyc*^0^ and *tim*^0^ mutants in constant light (LL). (F) In contrast, *Clk*^AR^ mutants exhibit an accelerated ARLI under constant light when compared to wild-type flies (ctrl) kept in LL as well. Graphs display the means ± SEM from 2–3 independent experiments.

We first examined the effects of genetic and environmental clock disruptions on *Drosophila* lifespan and ARLI, assessed by monitoring the performance, at successive ages, of groups of flies in a startle-induced negative geotaxis (SING) assay. We found that the *Clk*^AR^, *cyc*^0^ and *tim*^0^ mutations each had a similar significant but moderate impact on survival, reducing lifespan by at most 15% (S1A and S1B Fig and S1 Table). In *Drosophila*, ARLI was found to be faster in arrhythmic *per*^0^ mutants [8, 31]. Here we observed that ARLI was also moderately but significantly accelerated in the arrhythmic *tim*^0^ and *cyc*^0^ mutants, after 4 weeks of adult life (Fig 1A). Rearing Canton-S flies (controls) in LL, which stops the clock, produced a similar ARLI acceleration (Fig 1B and S1C Fig). This could be consistent with accelerated ARLI and reduced survival being general detrimental consequences of long-term circadian arrhythmicity.

ARLI in the arrhythmic *Clk*^AR^ mutant, however, was strikingly more precocious, starting as early as 10 days after adult eclosion (Fig 1C). Interestingly, locomotor decline was less rapid but still significantly faster, compared to control flies, in *Clk*^AR^/+ heterozygous flies, which are fully rhythmic [[32] and S2 Table], while it was not affected in *cyc*^0^/+ heterozygotes (Fig 1D). The impact of the *Clk*^AR^ mutation on ARLI thus appears separate from its effect on rhythmicity.

In contrast, average spontaneous locomotor activity in LD during the day displayed little change between young (10- to 15-day-old) and old (31- to 36-day-old) flies in any of the four tested genotypes (S1D Fig). In young *Clk*^AR^ and *cyc*^0^ flies, night-time activity was higher than in controls, as previously reported for *Clk*^AR^ [32], or than in *tim*^0^ mutants. Although night-time activity strongly decreased with age for both *Clk*^AR^ and *cyc*^0^ mutants, it did not become lower than in the controls (S1D Fig).

Circadian disruption also affects sleep, and sleep disruptions by themselves negatively impact the brain [33], and may thus contribute to accelerated ARLI. Sleep in LD was similarly affected in the *Clk*^AR^ and *cyc*^0^ mutants, with strongly increased latency after lights-off, and reduced total and night-time sleep (S2A–S2C Fig). These results are similar to those previously reported for *Clk*^Jrk^ and *cyc*^0^ [34]. However, the sleep profiles of the heterozygous mutants, *Clk*^AR^/+ and *cyc*^0^/+, were indistinguishable from controls (S2D Fig). In addition, knocking down *wake* (a gene involved in the transition from wake to sleep at the end of the day) in the LNvs, which was shown to disrupt sleep [34], did not affect the age-related impairment of the SING behavior (S2E Fig).

The *Clk*^AR^ mutation therefore produces a specific ARLI phenotype, which appears independent from its disrupting effect on circadian rhythmicity, and which does not correlate with accelerated global aging, disrupted sleep, or a major loss of motor ability.

### *Clk*^AR^ accelerates locomotor decline also in LL

In LL, ARLI was not significantly affected by the *cyc*^0^ or *tim*^0^ mutations (Fig 1E), suggesting that ARLI acceleration in these mutants is due to arrhythmia. In contrast, in LL ARLI was still much faster for the *Clk*^AR^ mutant than for control flies (Fig 1F). The difference between the *Clk*^AR^ mutant and the control in LL could be considered as representing the rhythm-independent effect of the *Clk*^AR^ mutation on ARLI.

Analyses of sleep showed no differences between controls and *Clk*^AR^ mutants in LL (S3A–S3C Fig). Indeed, in LL, total sleep time was similar for control, *Clk*^AR^ and *cyc*^0^ flies (S3B Fig). Sleep fragmentation, as assessed by average night (or presumptive night) bout duration, was stronger in LL than in LD (S3C Fig). However, in LL it was now very similar between all three strains. Thus the accelerated ARLI observed for the *Clk*^AR^ mutants in LL, relative to control or *cyc*^0^ flies, again does not correlate with a poorer sleep quality.

### *Clk* inactivation in PDF-expressing small LNv clock neurons accelerates ARLI

In order to determine where *Clk* function is required to maintain a wild-type SING behavior in aging flies, we used the *Gal4*-*UAS* system to express a *Clk* RNAi under the control of various *Gal4* drivers. Heterozygous *UAS-Clk*^RNAi^*/+* and *Gal4* driver*/+* flies were used as controls. When RNAi expression was driven in all clock cells with *tim-Gal4*, lifespan was significantly shortened relative to both controls (S4A Fig, S3 Table). ARLI was greatly accelerated in the *Clk*^RNAi^-expressing flies (S4B Fig). The acceleration was weaker than in the mutant, as it was not observed on days 10 and 17. This could only reflect a weaker loss of function, as *Clk*^RNAi^ also led to a weaker impact on free-running rhythms compared to *Clk*^AR^ (S2 Table). We also observed a smaller but significant effect on ARLI for the *tim-Gal4/+* control(S4B Fig).

**Fig 2:**
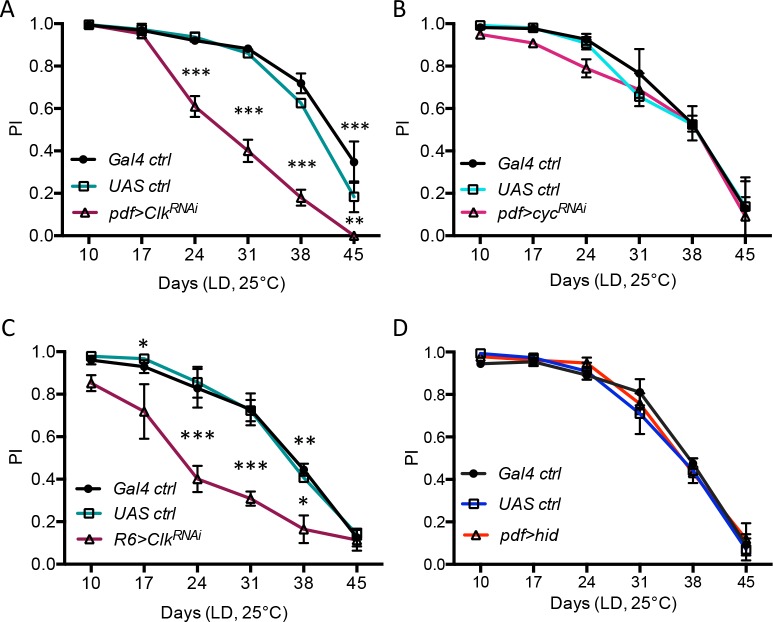
*Clk* inactivation in the s-LNv induces faster ARLI in aging flies. (A) Down-regulating *Clk* expression selectively in all PDF-expressing neurons (*pdf>Clk*^RNAi^) results in a premature locomotor decline. (B) Flies with reduced *cyc* expression in the PDF neurons (*pdf>cyc*^RNAi^) showed no ARLI defect. (C) Expressing *Clk*^RNAi^ in the s-LNv selectively (*R6-Gal4* driver) had the same effect on climbing abilities as *pdf>Clk*^RNAi^. (D) Ablation of PDF neurons by expressing the pro-apoptic gene *hid* in these cells did not alter ARLI. Graphs display the means ± SEM from 2 independent experiments.

When RNAi expression was driven selectively in PDF-expressing neurons with *pdf-Gal4*, there was no lifespan reduction (S4C Fig, S3 Table). However, ARLI was greatly accelerated, this time with the two controls behaving similarly to wild-type flies (Fig 2A). This indicates a specific effect of Clk on ARLI, independently of any acceleration in global aging. Accelerated ARLI was also observed with *pdf-Gal4* driving another independent *Clk*^RNAi^ (*UAS-Clk*^RNAi-R3^) (S4D Fig). Its weaker effect probably reflected a weaker inhibition of *Clk* function, as suggested by its also weaker effect on free-running rhythms (S2 Table). *cyc* knock-down in the LNvs had no effect on ARLI (Fig 2B), although *cyc*^RNAi^ disrupted behavioral rhythms more than *Clk*^RNAi^ (S2 Table). This is again in line with a specific effect of Clk deficiency on ARLI, unrelated to circadian rhythmicity. The *Clk*^AR^ mutation may lead to abnormal Clk protein products [32], but the similarity between the *Clk*^AR^ ARLI phenotype and that of the two *Clk* RNAi indicates that accelerated ARLI is likely due to Clk deficiency rather than a gain-of-function effect.

We used additional driver lines to further pin down the neurons where *Clk* function is required for wild-type ARLI in aging flies. Two neuronal groups express both PDF and Clk: the small LNvs (s-LNvs) and the large LNvs (l-LNvs). The *R6-Gal4* driver allowed us to knock-down *Clk* in the former, while the *C929-Gal4* driver allowed us to do the same in the latter. Knocking down *Clk* in the s-LNvs did accelerate ARLI (Fig 2C), while knocking down *Clk* in the l-LNvs had no effect (S4E Fig). Interestingly, we observed that ablating all the PDF neurons through expression of the pro-apoptotic gene *hid* [see [35]] also had no effect on ARLI (Fig 2D). This indicates that the s-LNvs are not required to preserve normal locomotor aging in *Drosophila* and that *Clk* deficiency may induce an alteration in the activity pattern of these cells that would lead to accelerated ARLI.

### *Clk* expression in the LNvs rescues the locomotor impairment of the *Clk*^AR^ mutant

**Fig 3:**
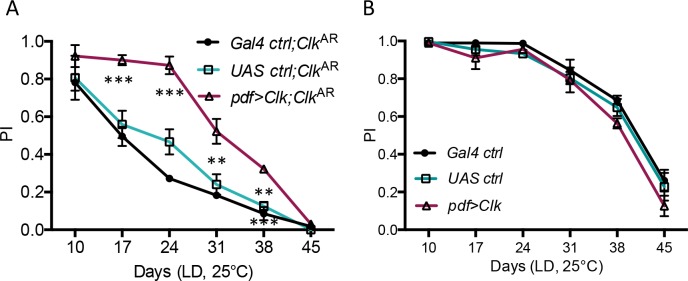
Rescue of *Clk*^AR^ ARLI phenotype by *Clk* expression in the PDF-expressing pacemaker neurons. (A) Reintroducing *Clk* expression in the PDF neurons of *Clk*^AR^ flies *(pdf>Clk;Clk*^AR^*)* significantly rescued the premature locomotor decline of the *Clk*^AR^ mutation. (B) *Clk* overexpression in PDF neurons had no effect on SING decline in a wild-type background. Graphs display the means ± SEM from 2 independent experiments.

We then asked conversely whether restoring *Clk* expression selectively in the PDF-expressing LNvs, using the *pdf-Gal4* driver, would rescue ARLI in an otherwise *Clk*^AR^ mutant background. This was indeed the case (Fig 3A), even though these rescued flies were behaviorally arrhythmic (S2 Table), as previously reported [32]. This further demonstrates a specific effect of Clk on ARLI that is unrelated to disruption of behavioral rhythms. We wondered whether overexpressing *Clk* in the LNvs would modulate ARLI in a wild-type background. It did not (Fig 3B), nor did it affect behavioral rhythms (S2 Table).

### ROS levels are increased in *Clk*^AR^ brains, but do not correlate with accelerated ARLI

**Fig 4:**
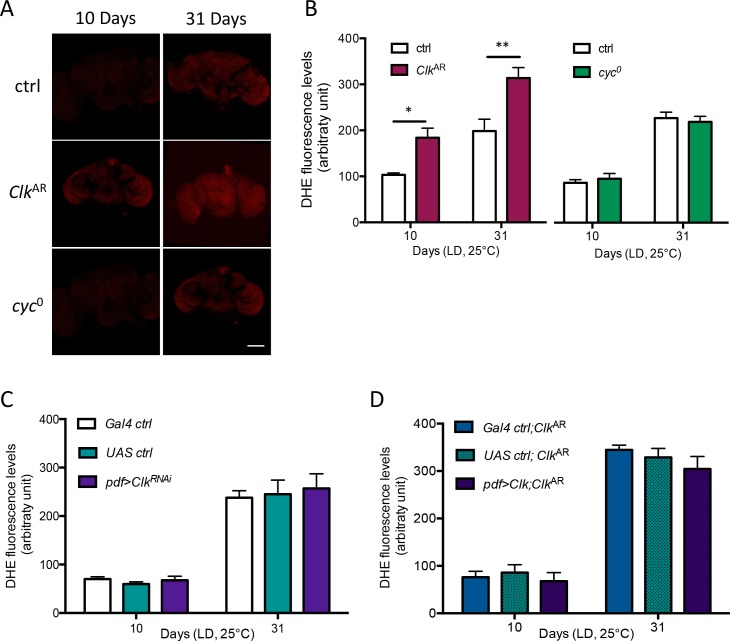
*Clk* regulates brain ROS levels independently from ARLI. (A, B) Brain ROS levels are higher in *Clk*^AR^ mutants at both 10 and 31 days of age as compared to ctrl and *cyc*^0^ flies. Scale bars in A: 100μm. (C) Downregulating *Clk* expression in the PDF neurons, which accelerated ARLI (see Fig 2A) had no effect on brain ROS levels as compared to controls. (D) Brain ROS levels of *Clk*^AR^ flies in which *Clk* expression was restored in the PDF neurons only, resulting in largely rescued ARLI (see Fig 3A), are unaltered in comparison to respective controls in the same *Clk*^AR^ background. Histograms display the mean ± SEM of brain ROS levels from 2–3 independent experiments with 6–8 brains per genotype in each experiment.

One factor that has been tentatively linked to ARLI is oxidative stress [19]. We therefore estimated brain ROS levels by dye staining in control, *Clk*^AR^ and *cyc*^0^ flies, at various ages post-eclosion. As previously reported for oxidative damage [8], ROS levels increase with age, especially between 24 and 31 days post-eclosion, and this was true for both control and *Clk*^AR^ flies (S5A Fig). This increase may play a part in the onset of ARLI in control flies, as it is concomitant with or slightly precedes it (Fig 1A, 1C and 1D). ROS levels were higher in *Clk*^AR^ than in controls already at 10 days, and remained higher at 31 days (Fig 4A and 4B and S5B Fig). By 45 days control and *Clk*^AR^ brains displayed similarly elevated ROS levels (S5B Fig). In contrast, no increase in brain ROS levels was found for the *cyc*^0^ mutant relative to control (Fig 4A and 4B). Increased ROS levels are then another *Clk*^AR^ mutant phenotype which, like strongly accelerated ARLI, is not caused by clock disruption alone.

The precociously elevated ROS levels in *Clk*^AR^ brains could be hypothesized to cause their precocious ARLI phenotype. However, we found no increase in ROS levels in the brains of *pdf>Clk*^RNAi^ flies relative to their two controls (Fig 4C), even at 31 days of age, although the former flies displayed accelerated ARLI well before that age (Fig 2A). Conversely, whereas restoring Clk expression selectively in the LNvs in a *Clk*^AR^ background rescued ARLI (Fig 3A), it did not reduce brain ROS levels relative to the two controls (Fig 4D). There is thus no correlation between elevated brain ROS levels and accelerated ARLI in Clk-deficient flies (*Clk*^AR^ and *pdf>Clk*^RNAi^), indicating that a global increase in brain oxidative stress is neither necessary nor sufficient to cause their accelerated ARLI phenotypes.

### *Clk* inactivation in the LNvs alters dopaminergic neurons in the PPL1 cluster, possibly via activation of apoptotic pathways

**Fig 5:**
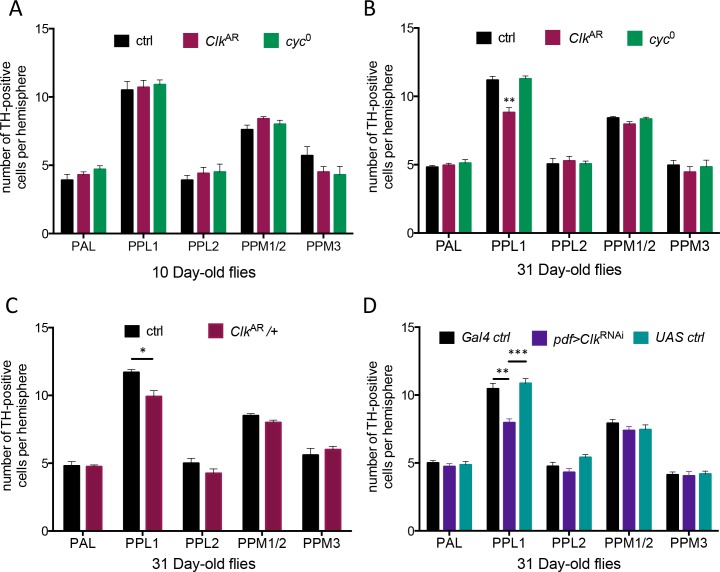
Specific loss of dopaminergic PPL1 neurons in *Clk*-deficient flies. TH-IR cells were counted in confocal stacks of brains dissected from flies of the indicated ages and genotypes. In all panels, histograms display the mean ± SEM of TH-IR cell numbers in the PAL, PPL1, PPL2, PPM1/2 and PPM 3 clusters, from 2 independent experiments with 8–10 brains per genotype in each experiment. (A) At 10 days of age, the number of TH-IR neurons in the different dopaminergic neuronal clusters of Canton-S (ctrl), *Clk*^AR^ and *cyc*^0^ flies was similar. (B) *Clk*^AR^ mutants exhibited a selective loss of TH-IR cells in the PPL1 neuronal cluster at 31 days post-eclosion when compared to either controls or *cyc*^0^. (C) Loss of TH-IR cells in the PPL1 neuronal cluster was also observed at 31 days post-eclosion in *Clk*^AR^/+ heterozygote flies. (D) Reducing *Clk* levels in the PDF neurons specifically decreased the number of PPL1 TH-IR cells. PAL, protocerebral anterior lateral; PPM1-3, protocerebral posterior median 1–3; PPL1-2, protocerebral posterior lateral 1–2.

Because dopaminergic circuits are involved in locomotor control in flies (see above in the Introduction), we wondered whether the *Clk*^AR^ mutation could affect dopaminergic neurons. The adult *Drosophila* brain contains eight clusters of dopaminergic neurons [36]. We counted the number of these neurons in most classes (S6 Fig) except the protocerebral anterior median (PAM) cluster, which was difficult to quantify precisely because of its large size. The number of neurons immunoreactive for tyrosine hydroxylase (TH-IR neurons), the rate-limiting enzyme for dopamine synthesis, was not significantly different in the brains of 10-day-old *Clk*^AR^, *cyc*^0^ and control flies (Fig 5A), at an age when the effect of the *Clk*^AR^ mutation on SING is still very small (see Fig 1C).

In contrast, in 31-day-old flies, we observed a significant and selective loss of TH-IR neurons in the PPL1 cluster of *Clk*^AR^ mutants relative to control, while *cyc*^0^ brains were unaffected (Fig 5B and S7 and S9A Figs). A similar loss was also observed in 31-day-old *Clk*^AR^/+ heterozygotes (Fig 5C and S7 Fig), as well as in *pdf*>*Clk*^RNAI^ flies where *Clk* expression was knocked down in the LNvs, relative to both controls (Fig 5D and S7 Fig). That loss, like the accelerated ARLI phenotype (Fig 3A), was rescued by Clk expression restricted to the PDF neurons (S10A Fig). In contrast, the size of several serotonergic clusters in a broad dorso-lateral protocerebral region [see [37]], where the PPL1 is located, did not differ between aging *Clk*^AR^ and control flies (S11 Fig). Accelerated ARLI due to impaired Clk function appears thus selectively associated with a loss of TH-IR neurons in the PPL1 cluster, although we cannot rule out that a few PAM neurons are also affected (see also below).

We observed that the PPL1 neuronal cell bodies are located close to the s-LNv dorsal projections, as they turn medially in the protocerebrum (S9A Fig), suggesting that these dopaminergic neurons could be influenced by the s-LNvs, through a direct or paracrine connection. Note that projections from the s-LNv appeared wild-type in the *Clk*^AR^, *Clk*^RNAi^- and *cyc*^RNAi^-expressing flies, while they were disrupted in *cyc*^0^ flies (S7–S9 Figs), as previously reported for both *cyc*^0^ and *Clk*^Jrk^ [38, 39]. Indeed one of the reasons that prompted us to analyze the *Clk*^AR^ rather than the *Clk*^Jrk^ mutant was to avoid such a developmental effect, another one being the known dominant-negative character of *Clk*^Jrk^ [40].

To test the possibility that PPL1 neuronal loss may involve the activation of apoptotic pathways, we used the H99 deficiency, which removes a pro-apoptotic gene cluster including *hid*, *rpr* and *grim*. H99/+ heterozygous flies are viable, but lack almost all embryonic programmed cell death [41]. No decrease the number of TH-IR PPL1 neurons was observed in H99/+, *Clk*^AR^ flies relative to H99/+ controls, which contrasted with *Clk*^AR^ alone (S10B Fig). Although this does not demonstrate that PPL1 neurons actually undergo apoptosis in aging *Clk*^AR^ brains, it indicates that the activation of apoptotic pathways is indeed involved in the mutant phenotype.

### *Clk*^AR^ age-related locomotor and dopaminergic phenotypes require the PDF receptor

**Fig 6:**
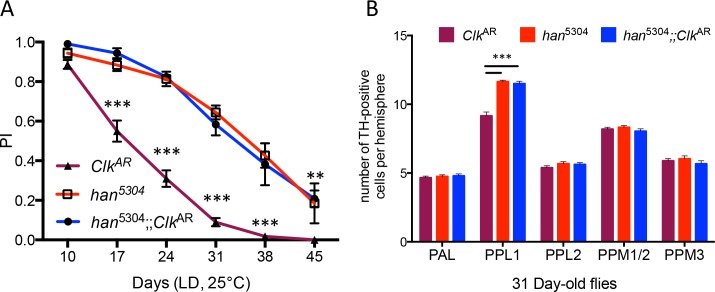
*Clk*^AR^ locomotor and neuronal phenotypes require the PDF receptor. (A) ARLI was assayed in the *Clk*^AR^ and *han*^5304^ mutants, and in the *han*^5304^;;*Clk*^AR^ double mutant. The latter did not show the accelerated ARLI characteristic of *Clk*^AR^ flies. The graph displays the means ± SEM from 2 independent experiments. (B) Histograms display the mean ± SEM of TH-IR cell numbers in the PAL, PPL1, PPL2, PPM1/2 and PPM 3 clusters, from 2 independent experiments with 7–8 brains per genotype in each experiment. At 31 days of age, no TH-IR cell loss was observed in either *han*^5304^ or *han*^5304^;;*Clk*^AR^, contrary to the loss of TH-IR cells in the PPL1 cluster characteristic of aged *Clk*^AR^ flies.

The main signaling molecule secreted by the s-LNv neurons is the neuropeptide PDF. It was recently reported that expression of a dominant-negative form of the circadian kinase *doubletime* (*dbt*^K/R^) in the LNvs led to transient daily caspase activation, prominently in the optic lobes, by a circadian-independent mechanism that requires PDF receptor signaling [42]. Transient caspase activation was also observed in head extracts of the *Clk*^Jrk^ mutant [42]. Here we found that the *Clk*^AR^ mutation was unable to accelerate ARLI in the absence of the PDF receptor (Fig 6A). Remarkably, in a *Clk*^AR^ mutant background, the lack of PDF receptor also fully rescued the loss of dopaminergic neurons in the PPL1 cluster (Fig 6B).

### Inhibiting TH expression in a single pair of PPL1 neurons accelerates ARLI

**Fig 7:**
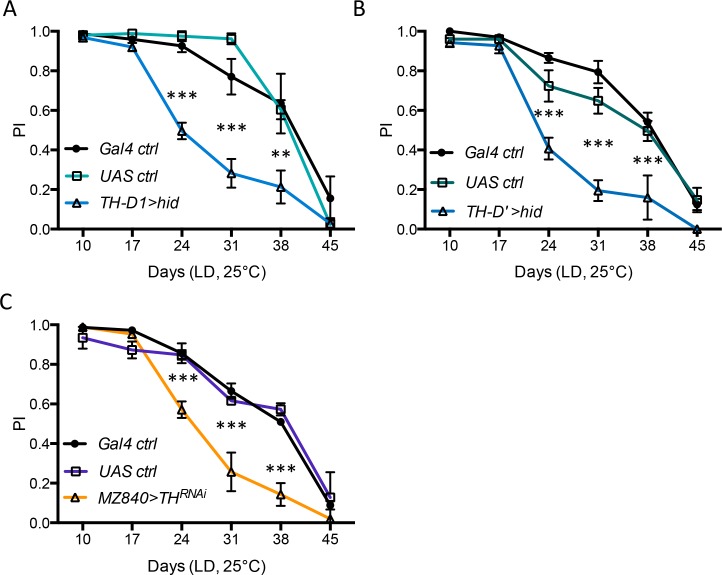
PPL1 dopaminergic neuron disruption accelerates ARLI. (A,B) Expression of the pro-apoptotic gene *hid* in dopaminergic neurons of the PPL1, PPL2 and PPM3 clusters with *TH-D1-Gal4* and *TH-D’-Gal4* drivers accelerates ARLI in aging flies. (C) *TH*^RNAi^ expression using *MZ840-Gal4* that targets one pair of dopaminergic neurons (the MB-V1 in each PPL1 cluster) as well as non-dopaminergic brain cells similarly speeded up SING decline with age. Data in panels A and B are from 2 separate experiments, with 5 groups of flies tested at each age for each genotype, the graphs showing means and sem for these 5 groups. Panel C is an average of 2 independent experiments with the indicated genotypes.

To test the possibility that loss of TH-IR neurons in the PPL1 cluster may impair SING in older flies, we first eliminated at least part of these neurons by expressing the pro-apoptotic gene *hid* under control of the *TH-D1-Gal4* driver [43]. This accelerated SING decline (Fig 7A), comparably to the effect of knocking down *Clk* in the s-LNvs (Fig 2A). Similar results were obtained with the *TH-D'-Gal4* driver (Fig 7B), which is more specific for dopaminergic neurons [43]. However, *TH-D'-Gal4* targets other dopaminergic neurons, outside the PPL1, in the PPL2 and PPM3 clusters, which may contribute to the observed effect on ARLI. Therefore we used an alternative strategy [27]: inactivating *Drosophila TH* by targeted RNAi. By driving *TH* RNAi expression with *MZ840-Gal4*, which is expressed in only one PPL1 neuron (MB-V1) and in other non-dopaminergic brain cells [44], the two MB-V1 neurons should be the only affected ones. We observed that *TH* knock-down with *MZ840-Gal4* also accelerated ARLI (Fig 7C), again comparably to the effect of *Clk* knock-down in the s-LNvs (Fig 2A). The impact of reducing Clk function in the s-LNvs is thus phenocopied by reducing the number or function of PPL1 neurons, and particularly of the MB-V1 pair, consistent with other neurons (including dopaminergic ones within the PAM) not playing a major effect in the accelerated ARLI of *Clk*^AR^ flies.

## Discussion

### A circadian-independent neuroprotective function of Clk in the LNvs?

We report here that both the *Clk*^AR^ mutation and reducing Clk function in the PDF-expressing LNvs led to faster decline of a startle-induced locomotor response in aging flies, and to dopaminergic neuron disruption selectively in the PPL1 cluster. A significant acceleration of ARLI was also observed in heterozygous *Clk*^AR^/+ flies, which are fully rhythmic. Such *Clk* gene-specific phenotypes were indeed completely clock-independent, as they were not observed when the circadian clock was stopped or perturbed in other ways, such as in LL conditions, or by *cyc*^0^ and *tim*^0^ mutations. In contrast, we found that spontaneous locomotor activity, sleep, and lifespan were similarly affected in the *Clk*^AR^ and *cyc*^0^ mutants.

Other clock-independent functions of clock genes or the LNvs have been reported in *Drosophila*, e.g. in reproduction [45, 46], responses to cocaine [47, 48] and sensitivity to sleep deprivation [49]. In the latter case, Cyc seems to play a specific role, as *Clk*, *per*, and *tim* mutants do not display hypersensitivity to sleep deprivation like the *cyc*^0^ mutant. In what cells Cyc is required to protect the flies from the effects of sleep deprivation is not known. However, in most studies, *Clk* and *cyc* mutants had the same or similar phenotypes [34, 40, 50–53]. Clk and Cyc are co-expressed in all brain clock neurons, and neither one appears to be expressed in any other brain neurons [54]. Ectopic expression of Clk but not Cyc is sufficient to induce a functional clock in other brain neurons; it does require Cyc function, however [55, 56]. It remains to be investigated whether Clk requires another partner in circadian neurons to prevent accelerated ARLI and dopaminergic neuron loss.

We observed that removing the PDF-expressing neurons had no effect on ARLI, in contrast to the striking phenotype induced by *Clk* inactivation in the same neurons. This suggests that *Clk* inactivation does not inhibit but rather overactivates PDF-neuron signaling. Both the locomotor and neuronal loss phenotypes of the *Clk*^AR^ mutant were indeed rescued in the absence of PDF receptor. Recently, the *Clk*^Jrk^ mutation, but not *per*^0^, was shown to transiently activate the Dronc caspase in fly heads, in response to light during the day. Furthermore, the expression of a dominant negative form of the circadian kinase *doubletime* (*dbt*^K/R^) in the LNvs was shown to activate the Dronc caspase in large parts of the brain, independently of the clock itself but dependent on PDF receptor signaling [42]. It is therefore quite possible that *Clk* inactivation in the s-LNvs can induce PDF receptor-dependent caspase activation in target or neighbouring cells, that will eventually lead, directly or indirectly, to dopaminergic neuron loss (see below).

The precocious increase in ROS levels in *Clk*^AR^ mutant brains could also contribute to caspase activation. Again, the clock and Cyc do not seem to be involved as ROS levels were not affected in the *cyc*^0^ mutant. However, our targeted *Clk* rescue and knock-down experiments suggest that a global increase in brain ROS levels accelerates ARLI only marginally, if at all, in this context. The global ROS increase may contribute to some differential effects of the *Clk*^AR^ versus *cyc*^0^ mutations (e.g. on sensitivity to sleep deprivation, since elevated ROS levels may induce protective mechanisms, see e.g. [57]), or of the *Clk*^AR^ mutation versus LNv-restricted expression of *Clk*^RNAi^ (such as the one we found on longevity).

### A PDF-receptor-dependent link between the LNvs and a specific dopaminergic cluster

**Fig 8:**
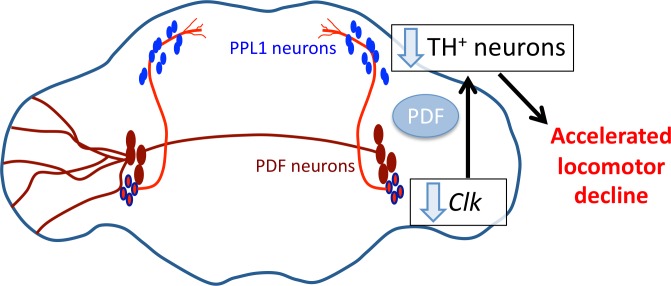
Proposed link between PDF-expressing and dopaminergic neurons in the *Drosophila* brain. Reducing *Clk* expression in the s-LNv neurons (bright red with blue outline) accelerates ARLI, and perturbates the dopaminergic PPL1 cluster (somas shown in blue). The PDF receptor is involved, possibly overactivated by PDF released from the s-LNvs. In the *Clk*^AR^ mutant, this could in particular negatively affect the MB-V1 dopaminergic neuron, either directly or indirectly, leading to accelerated ARLI. The projections of the l-LNv neurons (brown red) onto the right brain hemisphere were omitted for clarity.

Our results suggest the existence of a link between the s-LNvs and the PPL1 cluster of dopaminergic neurons that involves PDF-receptor signaling (Fig 8). Inverse links between clock and DA neurons have already been demonstrated: dopamine strongly modulates cAMP levels in l-LNvs, via different receptor subtypes, while s-LNvs are much less responsive [58]. The identity of the DA neurons afferent to the l-LNvs is still unknown.

We observed that the PPL1 cluster lays close to the s-LNv projections to the dorsal protocerebrum. s-LNv projections were impaired in the *cyc*^0^ mutant, as previously shown [38, 39], but not in the *Clk*^AR^ mutant. This difference does not seem to play a part in the differential impact of the two mutants on ARLI, since s-LNv projections appeared completely wild-type when expressing RNAi for either *cyc* or *Clk* in the PDF neurons. Although *cyc*^RNAi^ was more effective than *Clk*^RNAi^ in disrupting circadian activity rhythms, it did not accelerate ARLI at all. As no PDF processes were observed in apposition to TH-IR neurons in the posterior dorsal brain [59], signaling to the PPL1 may well be paracrine rather than synaptic, consistent with both anatomical [60] and functional [61] evidence for such signaling mechanism by PDF. On the other hand, PDF receptor in that protocerebral region appears exclusively expressed in 3 LNd clock neurons, as judged from anti-MYC labeling of a PDF receptor-MYC fusion [62]. The LNds might thus be intermediary neurons mediating the influence of the s-LNvs on the PPL1.

What happens to the PPL1 neurons when Clk function is compromised in the LNvs? Similar questions were raised in fly models of PD, where the size of various TH-IR neuronal clusters often appeared reduced (see e.g. [63–68]), but actual cell loss was sometimes contested [69, 70]. Here we report that reducing TH levels in specific neurons was sufficient to accelerate ARLI, as was also shown in our previous study [27]. In the latter, accelerated ARLI was attributed not to cell loss, but to age-related alterations in contacts onto the mushroom bodies from a subset of ~15 PAM neurons, when they express α-synuclein (a protein involved in PD). In agreement with that, we found that locomotion was already affected in 10-day-old *Clk*^AR^ mutants, before any visible reduction in dopaminergic cell numbers. The MB-V1 neuron, which is currently our best candidate among PPL1 neurons as mediator of Clk deficiency-induced ARLI acceleration, also projects to the mushroom bodies [71]. It remains to be seen whether this specific neuron is among those that disappear or lose TH-IR in aging flies when *Clk* is down-regulated in the LNvs, and whether the loss of other PPL1 neurons would be sufficient to accelerate ARLI.

The PPL1 cluster was reported to be selectively reduced in flies expressing α-synuclein under control of *TH-Gal4* [66], a driver that does not express strongly in the PAM [23]. That reduction was rescued in an apoptosis-deficient genetic background [66], similarly to what we observed for the effect of the *Clk*^AR^ mutation, or by overexpression in dopaminergic neurons of either glutathione S-transferase Gst1 [66], the Nrf2 transcription factor [67] or the endosomal recycling factor Rab11 [72]. Whether these latter effectors can also protect the PPL1 neurons in Clk-deficient flies, as well as how apoptotic pathways may affect PPL1 neurons in these contexts, remain open questions.

### Requirement of the Clk protein for dopaminergic circuits in flies and mice

Our results show that *Clk* inactivation in the main *Drosophila* pacemaker neurons, the s-LNvs, has dramatic consequences for a locomotor response and select dopaminergic neurons maintenance in aging flies, independently of the less prominent but significant defects induced by circadian rhythm disruptions. Such progressive, circadian clock-independent effects both add to, and contrast with, previously described links between Clk and dopaminergic signaling. The acute nocturnal hyperactivity and reduced sleep of *Clk*^Jrk^ mutants, for instance, were attributed to increased dopaminergic transmission [73]. However, they involved Cyc as well, and the l-LNvs rather than the s-LNvs. In mice, a *Clk* mutant also displays decreased sleep and hyperactivity, as well as a mania-like behavior, presumably owing to increased activity of dopaminergic neurons in the ventral tegmental area [74, 75]. However, contrary to flies, *Clk* is expressed in such neurons, where its loss increases expression of genes involved in dopaminergic signaling, including TH [74, 75], consistent with the observed phenotypes.

Interestingly, the combined deletion of *Clk* and its paralog *Npas2* induced severe age-dependent astrogliosis in the mouse brain, leading to degeneration of synaptic terminals, neuronal oxidative damage and impaired expression of several redox defense genes [76]. Neuron and glia-specific inactivation of BMAL1, the mouse ortholog of Cyc, produced similar phenotypes. In *Drosophila*, our results now indicate that *Clk* regulates s-LNv activity, independently of its role in the circadian machinery, and that the loss of this regulation leads to progressive dysfunction of specific brain dopaminergic neurons, by mechanisms that involve PDF receptor signaling. Further deciphering the wide-ranging effects of *Drosophila* Clk dysfunction, whether circadian clock-dependent or not, or cell-autonomous or not, could shed new light on the regulation of dopaminergic neuron survival in both flies and mice.

## Materials and Methods

### *Drosophila* culture and strains

Flies were maintained on standard medium at 25°C, under a 12:12 Light/Dark cycle (LD 12:12), or under constant light. Light intensity at the level of the flies was in the range 500–3000 lux. The following clock mutants were backcrossed for five generations to a wild-type (Canton-S) genetic background before use: *Clk*^AR^ [32], *cyc*^0^ [77], *pdf*^0^ [35], *tim*^0^ [78]. *cyc*^0^, *pdf*^0^ and *tim*^0^ are null mutations, whereas *Clk*^AR^ is a strong hypomorph that is behaviorally arrhythmic but with detectable Per oscillations in peripheral tissues [32]. Other strains included the PDF receptor mutant *han*^5304^ [79], the H99 deficiency (Bloomington *Drosophila* Stock Center (BDSC) strain #1576) and the following *Gal4* drivers: *C929-Gal4* [80], *MZ840-Gal4* [81], *pdf-Gal4* [35], *R6-Gal4* [82], *TH-D’-Gal4 and TH-D1-Gal4* [43], *tim-Gal4* [83] and *UAS* strains: *UAS-Clk-B19* (C. Michard-Vanhée, B. Richier and F. Rouyer, personal communication), *UAS-Clk*^RNAi^ (TriP HMJ02224, BDSC #42566) [84], *UAS-Clk*^RNAi-R3^ (7391R-3 strain, National Institute of Genetics, Japan), *UAS-cyc*^*RNAi*^ (TriP HMJ02219, BDSC #42563), *UAS-hid* [85], *UAS-TH*^RNAi^ (TriP JF01813, BDSC #25796), *UAS-Wake*^*miR1*^ [34]. H99, *Clk*^AR^ recombinant chromosomes were obtained via standard *Drosophila* genetics. As they are homozygous lethal, like the H99 parental chromosome, they were identified by assaying the rhythmicity of progeny from crosses between H99 *Clk*^AR^ / TM3(Sb) and *Clk*^AR^ flies.

### Locomotor activity rhythm monitoring

Locomotor activity experiments were performed using commercial activity monitors (TriKinetics) placed in incubators equipped with standard white, fluorescent low-energy tubes. Young (10 day-old male flies) or older (30 day-old males) males were maintained 5–6 days under LD 12:12 (Light-Dark 12h:12h), and then switched to at least 5–6 days of constant darkness, all on 5% sucrose-agar medium at 25°C. Data analysis was performed with the FaasX software, as described previously [86]. For constant darkness experiments, analysis started the second day of constant darkness. Histograms represent the distribution of the activity through 24 h in 30 min bins, averaged for *n* flies over 4–5 cycles. All behavioral experiments were repeated 2–3 times to verify reproductibility.

### Sleep monitoring

3–4 days old male flies were placed individually in Trikinetics glass tubes, video monitored under infra-red illumination during 3 days in LD, then placed for at least 5 days in LL. The images were processed using pySolo video software [87] to determine the distance travelled (in pixels) for each minute of the day. Sleep was defined as 5 min of more of immobility [88]. At least 40 flies in 2–3 replicates were analyzed for each genotype.

### Lifespan assay

Male flies were maintained on standard medium at 25°C and under either LD 12:12, or constant light from adult day 1 until death. 50 animals/bottle in triplicate were tested for each genotype in a given experiment, and each experiment was performed at least twice.

### Locomotor assay

Male flies were aged under the same conditions as for the lifespan assays. SING assays were performed as described previously [27, 64]. Groups of 10 flies were placed in a vertical column (25 cm long, 1.5 cm diameter) with a conic bottom end and left for about 20–25 min for habituation. Then, for each genotype, 5 columns were tested individually by gently tapping down the flies (startle), which normally respond by climbing up. Each fly group was assayed three times at 15 min intervals.

Results are the mean and SEM of the scores obtained with the 5 independent groups of flies per genotype. The performance index (PI) for each column was calculated as follows: ½[1 + (n_top_-n_bot_)/n_tot_], where n_tot_ is the total number of flies in the column, n_top_ is the number of flies that have reached at least once the top of the column (above 22 cm) during a 1 min interval, and n_bot_ is the number of flies that never left the bottom (below 4 cm). ARLI was monitored as described previously by testing SING performance weekly over 6 weeks, starting on day 10 after eclosion [27]. Dead flies were replaced by substitutes of the same age. Experiments were repeated 2 to 3 times at different periods of the year.

### Immunohistochemistry

Immunostaining was performed essentially as previously described [26]. Adult flies of the desired ages were briefly washed in 70% ethanol before brain dissection in ice-cold *Drosophila* Ringer Ca^2+^ free solution. Brains were then fixed for 1h with shaking at room temperature in fixative containing 4% paraformaldehyde in PBS. Brains were then washed 3 x 20 min with PBS and incubated overnight in PBS/0.5% Triton X-100 + 2% BSA, at 4°C. Staining with primary antibody was carried out overnight at 4°C in PBS/0.5% Triton X-100 + 2% BSA. Brain were then washed 3 x 20 min with PBS/0.5% Triton X-100, and incubated for 2h at room temperature with secondary antibody diluted in PBS/0.5% Triton X-100 + 2% BSA. Finally, 3 x 20-min washes were performed using PBS.

Primary antibodies used included: mouse monoclonal anti-TH (Immunostar, 1:1000), rabbit polyclonal anti-PDF (gift from F. Rouyer's lab, 1:1000), rabbit polyclonal anti-5-HT (Sigma, 1:1000). Secondary antibodies included: anti-mouse or anti-rabbit conjugated to Alexa Fluor 488 or 555 (Invitrogen Molecular Probes, 1:1000).

### ROS measurement

*In situ* ROS detection was performed using a dihydroethidium (DHE) dye (Life technologies) following a previously described protocol [89] we adapted to whole-mount *Drosophila* brains. Briefly, male flies were aged under the same conditions as for lifespan assays and dissected in Schneider’s insect medium. The brains were then incubated with DHE for 5 minutes in the dark, fixed for 5 minutes in 7% formaldehyde in 1 X PBS, and immediately imaged on a confocal microscope, as indicated below. Relative ROS levels were measured by quantification of the dye fluorescence using the Fiji software [90].

### Fluorescent microscopy

Fly brains were mounted on slides using as antifade reagent either Prolong Gold (Life Technologies), for brain immunostaining, or Vectashield (Vector Laboratories), for ROS measurements. Brains were visualized and images acquired with a Nikon A1R confocal microscope. A minimum of 10–15 brains was scored over at least 2 trials. Laser, filter and gain settings remained constant within each experiment, and channels were scanned sequentially. Confocal Z-stacks were analyzed using ImageJ software: dopaminergic cells were counted for each cluster, and whole brain average intensity levels were measured for ROS detection.

### Statistics

For lifespan assays, survival curves were generated and compared using the log-rank (Mantel-Cox) test, and 150 animals were tested per genotype, with each experiment performed 2–3 times. For SING assays and fluorescence quantification, the mean and SEM were calculated for each trial and two-way Anova with post-hoc Tukey comparisons was used. Staining data were analyzed by one-way Anova and Tukey’s pairwise comparisons. Statistical analysis of sleep was performed using the Kruskal-Wallis test. GraphPad Prism 6 was used for all statistical analyses. Significant values in all figures: *: p<0.05, **: p<0.01, ***: p<0.001.

## Supporting Information

S1 Fig Lifespan, ARLI and activity rhythms of clock mutants. (A,B) Lifespan was comparably shortened in arrhythmic *cyc*^0^, *tim*^0^ (A) and *Clk*^AR^ flies (B) in LD. (C) SING decline was similar in the arrhythmic *cyc*^0^ and *tim*^0^ mutants in LD, and in the controls in LL. (D) In LD, average spontaneous locomotor activity during the day was not altered between young (10- to 15-day-old) and old (31- to 36-day-old) flies, for any genotype tested. Night time activity in young *Clk*^AR^ and *cyc*^0^ mutants was much higher than in controls, but it decreased with age for both mutants. Total activity was also higher in young *Clk*^AR^ and *cyc*^0^ mutants, as compared to control flies. The histograms show the mean and sem from two independent experiments, each of which included 24–32 flies for each age and genotype.
(PDF)

S2 Fig Sleep profiles of *Clk*^AR^ and *cyc*^0^ mutants in LD. Sleep architecture was similarly affected in *Clk*^AR^ and *cyc*^0^ mutant flies in LD (A, B, C). (A) Sleep in min/hour during a typical 24h LD day: *Clk*^AR^ and *cyc*^0^ flies showed delayed sleep onset and reduced sleep during the dark period. (B) Latency (time interval between lights off and the initiation of the first sleep bout of the dark period) was dramatically extended in *Clk*^AR^ and *cyc*^0^. (C) Total sleep was similarly reduced in *Clk*^AR^ and *cyc*^0^ compared to controls, due to the low sleep quotas during the dark. (D) Sleep architecture was not altered in *Clk*^AR^/+ and *cyc*^0^/+ heterozygote mutants when compared to Canton-S (ctrl) flies. (E) Inactivation of *wake* in PDF neurons is known to disrupt sleep [34] but it did not affect ARLI as monitored by SING behavior.
(PDF)

S3 Fig Sleep profiles of *Clk*^AR^ and *cyc*^0^ mutants in LL. (A) In LL, both control, *Clk*^AR^ and *cyc*^0^ flies lose sleep rhythms. Total sleep (B) and sleep bout duration (C) were not significantly different between genotypes. (C) Compared to LD, average sleep bout during the presumptive night was reduced for controls and *cyc*^0^ in LL.
(PDF)

S4 Fig Lifespan and ARLI of flies expressing *Clk*^RNAi^ in clock cells. *Clk*^RNAi^ expression in all clock cells (*tim*>*Clk*^RNAi^) decreases longevity (A) and strongly accelerates ARLI (B). Note that ARLI of the *tim-Gal4*/+ control is also impaired. (C) Expression of *Clk*^RNAi^ in PDF neurons does not affect longevity. (D) Using a second *Clk*^RNAi^ (*UAS-Clk*^RNAi-R3^) to inactivate *Clk* in PDF neurons also impairs locomotor performances. (E) Expressing *Clk*^RNAi^ in the l-LNv (*C929>Clk*^RNAi^) had no consequence on SING decline.
(PDF)

S5 Fig Brain ROS levels in adult *Clk*^AR^ flies. (A) Brain ROS increase with age between days 24 and 45 post-eclosion in control flies and between days 24 and 31 in *Clk*^AR^ mutants. (B) *Clk*^AR^ flies exhibit higher brain ROS than controls from day 10 post-eclosion to day 31. Both panels show the same data arranged differently to facilitate comparisons.
(PDF)

S6 Fig Localization of the TH-IR neuronal clusters analyzed in the *Drosophila* brain. The hemisphere on the left corresponds to an anterior view, the right one to a posterior view. Approximate positions of esophagus and calyx of mushroom body are shown to provide orientation cues. On the left, only the approximate position of the large PAM (protocerebral anterior median) cluster is indicated, as we did not attempt to count its more than 100 neurons [36]. Other abbreviations: PAL, protocerebral anterior lateral; PPM1-3, protocerebral posterior median 1–3; PPL1-2, protocerebral posterior lateral 1–2.
(TIF)

S7 Fig PDF and TH neuronal patterns in circadian mutant brains. Co-immunostainings against TH (*green*) and PDF (*magenta*) of whole-mount adult brains of aged Canton-S flies (ctrl) (31 days post-eclosion). *Clk*^AR^, *Clk*^AR^/+ and pdf>*Clk*^RNAi^ exhibit normal s-LNv dorsal projections (PDF, *magenta*) but reduced TH-positive cells (TH, *green*) in the PPL1 cluster (boxed region in ctrl brain, see also S7A and S7B Fig). s-LNv dorsal projections are altered in *cyc*^0^ mutants. Scale bar: 100μm.
(PDF)

S8 Fig Higher magnification of PDF projection profiles in circadian mutant brains. A different sample of the same genotypes shown in S7 Fig. They illustrate that s-LNv dorsal projections are reduced only in the *cyc*^0^ background. Scale bars: 100μm (left panels), 50μm (right panels).
(PDF)

S9 Fig Projections of the s-LNvs pass close to the PPL1 dopaminergic cluster. (A) Co-immunostainings against TH (*green*) and PDF (*magenta*) of whole-mount brains of 31-days-old flies at higher magnification, showing that PPL1 TH-positive cells (*green*) are localized in the vicinity of the s-LNv dorsal projection (*magenta*). The number of TH-positive cells is reduced in the *Clk*^AR^ brain (right panel) as compared to the control brain (left panel). (B) *cyc*^RNAi^ expression in the PDF neurons does not disrupt s-LNv dorsal projections. Scale bars: (A) 25μm, (B) 100μm (left panel), 50μm (right panel).
(PDF)

S10 Fig **The number of PPL1 TH-IR neurons in the *Clk***^**AR**^ **mutant is rescued by restoring Clk expression in the PDF neurons (A), or by inhibiting apoptosis (B).**TH-IR cells were counted in confocal stacks of brains dissected from flies of the indicated genotypes. Bars display the mean ± SEM of TH-IR PPL1 cell numbers, from 2 independent experiments, each with 8–10 brain hemispheres per genotype. The age of the dissected flies was 31 days in one experiment, and 35–36 days in the other. Although there may be a small effect of the H99 deficiency by itself, the *Clk*^AR^ mutation has clearly no effect on the number of PPL1 neurons in the presence of that deficiency.
(PDF)

S11 Fig Serotonergic clusters located close to the PPL1 region are not affected in the *Clk*^AR^ mutant. TH-IR and 5-HT-IR cells were counted in confocal stacks of brains dissected from 31-day-old control and *Clk*^AR^ flies. (A) Bars display the mean ± SEM of TH-IR (PPL1) and 5-HT-IR (PLP, LP and ALP) cell numbers, from 8–10 brain hemispheres per genotype. ALP: Anterior Lateral Protocerebrum, LP: Lateral Protocerebrum, PLP: Posterior Lateral Protocerebrum [37]. (B) z-projections of the anterior and posterior parts of a representative 31-day-old *Clk*^AR^ brain, double-labeled with anti-TH and anti-5-HT antibodies, as indicated. Scale bar: 100μm.
(PDF)

S1 Table Longevity of *Clk*^AR^ mutants (related to S1A and S1B Fig). (DOCX)

S2 Table Rest-activity rhythms in DD at 25°C. (DOCX)

S3 Table Longevity of flies expressing *Clk*^RNAi^ in clock cells (related to S4A and S4C Fig). (DOCX)
