# Supplementary figures and images for: Drosophila Clock Is Required in Brain Pacemaker Neurons to Prevent Premature Locomotor Aging Independently of Its Circadian Function
Source: PLoS Genet. 2017 Jan 10;13(1):e1006507. doi: 10.1371/journal.pgen.1006507 (PMC5224980; doi:10.1371/journal.pgen.1006507)

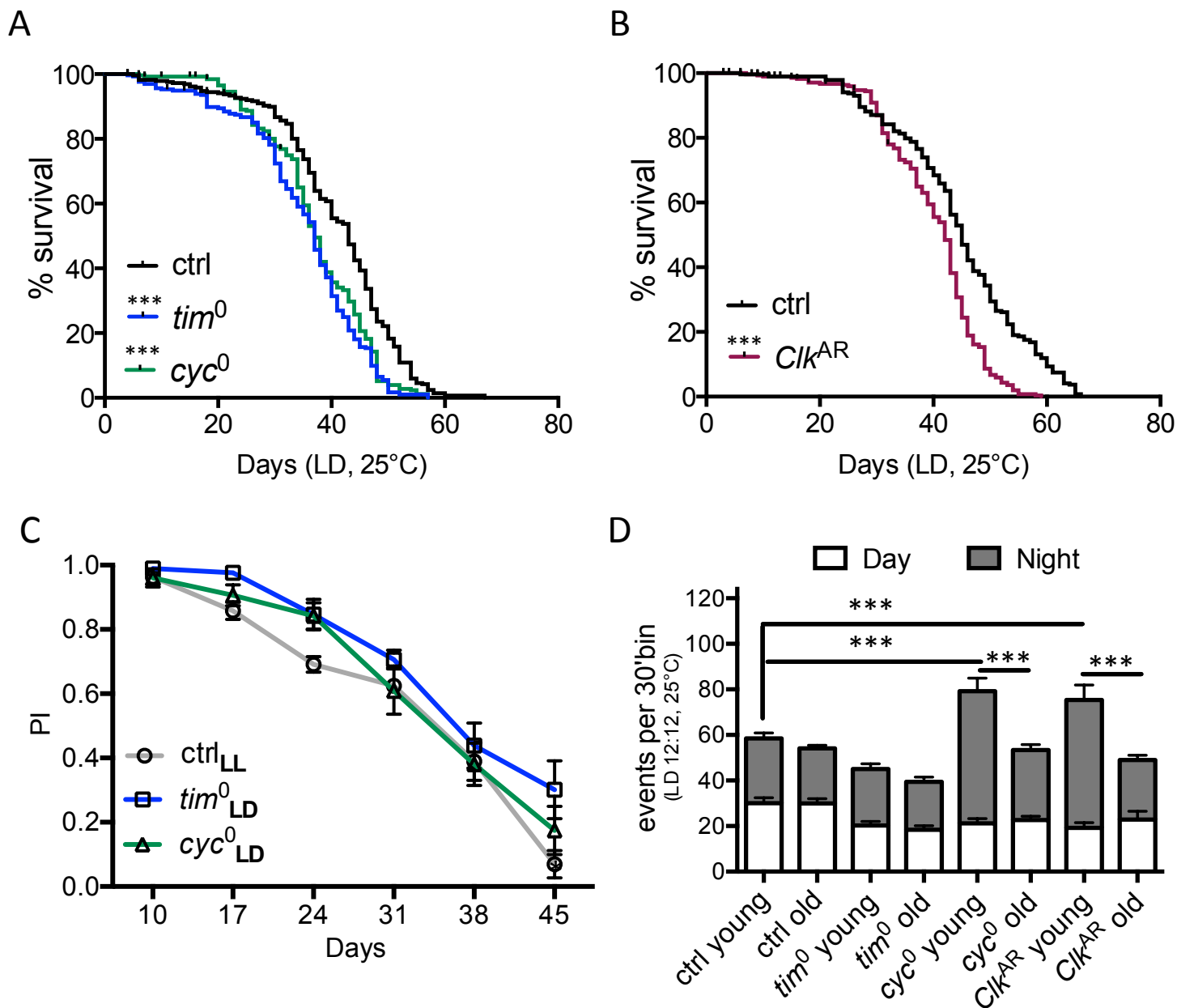

Figure S1

Supplement: S1 Fig — (A,B) Lifespan was comparably shortened in arrhythmic cyc0, tim0 (A) and ClkAR flies (B) in LD. (C) SING decline was similar in the arrhythmic cyc0 and tim0 mutants in LD, and in the controls in LL. (D) In LD, average spontaneous locomotor activity during the day was not altered between young (10- to 15-day-old) and old (31- to 36-day-old) flies, for any genotype tested. Night time activity in young ClkAR and cyc0 mutants was much higher than in controls, but it decreased with age for both mutants. Total activity was also higher in young ClkAR and cyc0 mutants, as compared to control flies. The histograms show the mean and sem from two independent experiments, each of which included 24–32 flies for each age and genotype. (PDF) [file pgen.1006507.s001.pdf]

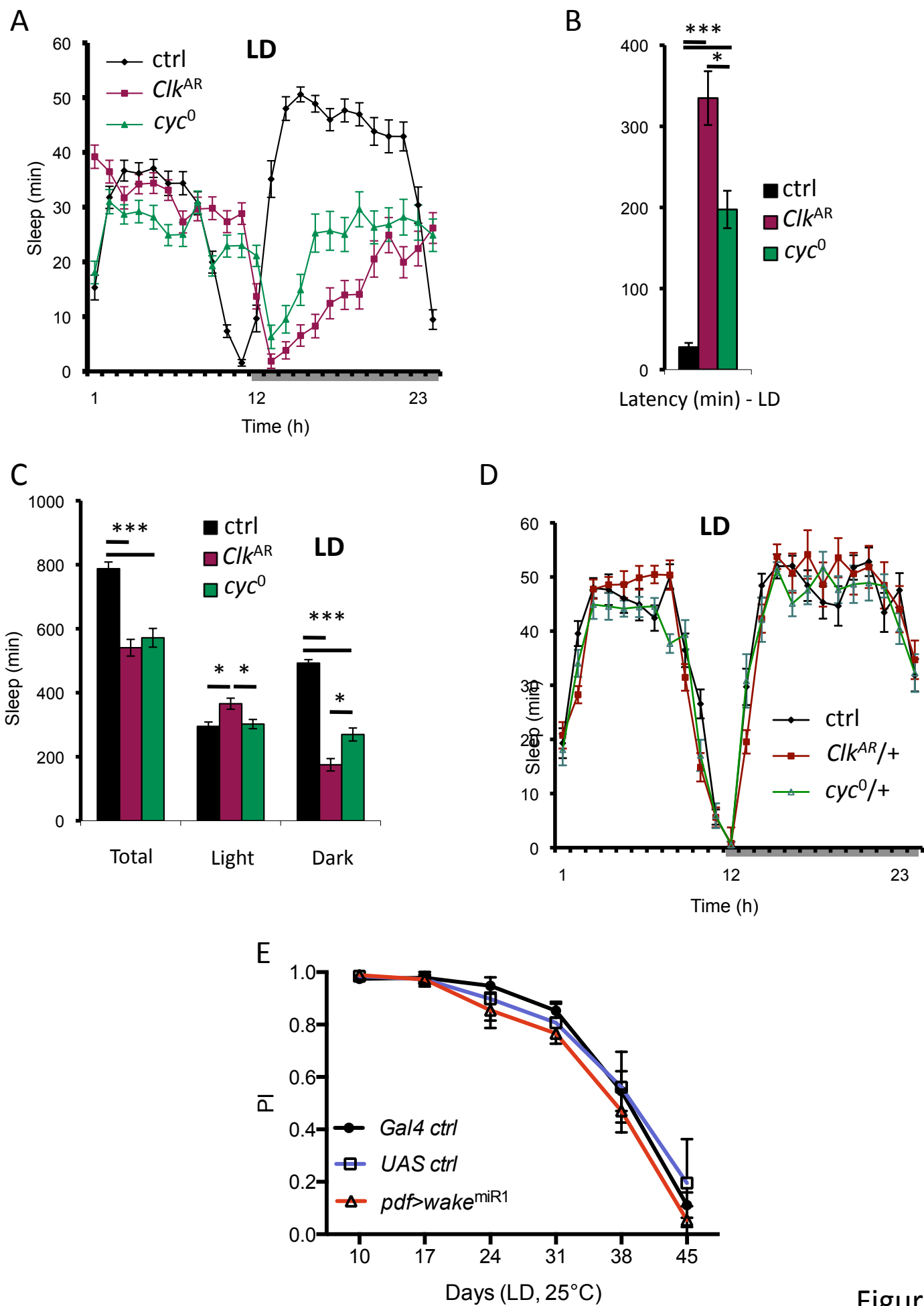

Figure S2

Supplement: S2 Fig — Sleep architecture was similarly affected in ClkAR and cyc0 mutant flies in LD (A, B, C). (A) Sleep in min/hour during a typical 24h LD day: ClkAR and cyc0 flies showed delayed sleep onset and reduced sleep during the dark period. (B) Latency (time interval between lights off and the initiation of the first sleep bout of the dark period) was dramatically extended in ClkAR and cyc0. (C) Total sleep was similarly reduced in ClkAR and cyc0 compared to controls, due to the low sleep quotas during the dark. (D) Sleep architecture was not altered in ClkAR/+ and cyc0/+ heterozygote mutants when compared to Canton-S (ctrl) flies. (E) Inactivation of wake in PDF neurons is known to disrupt sleep [34] but it did not affect ARLI as monitored by SING behavior. (PDF) [file pgen.1006507.s002.pdf]

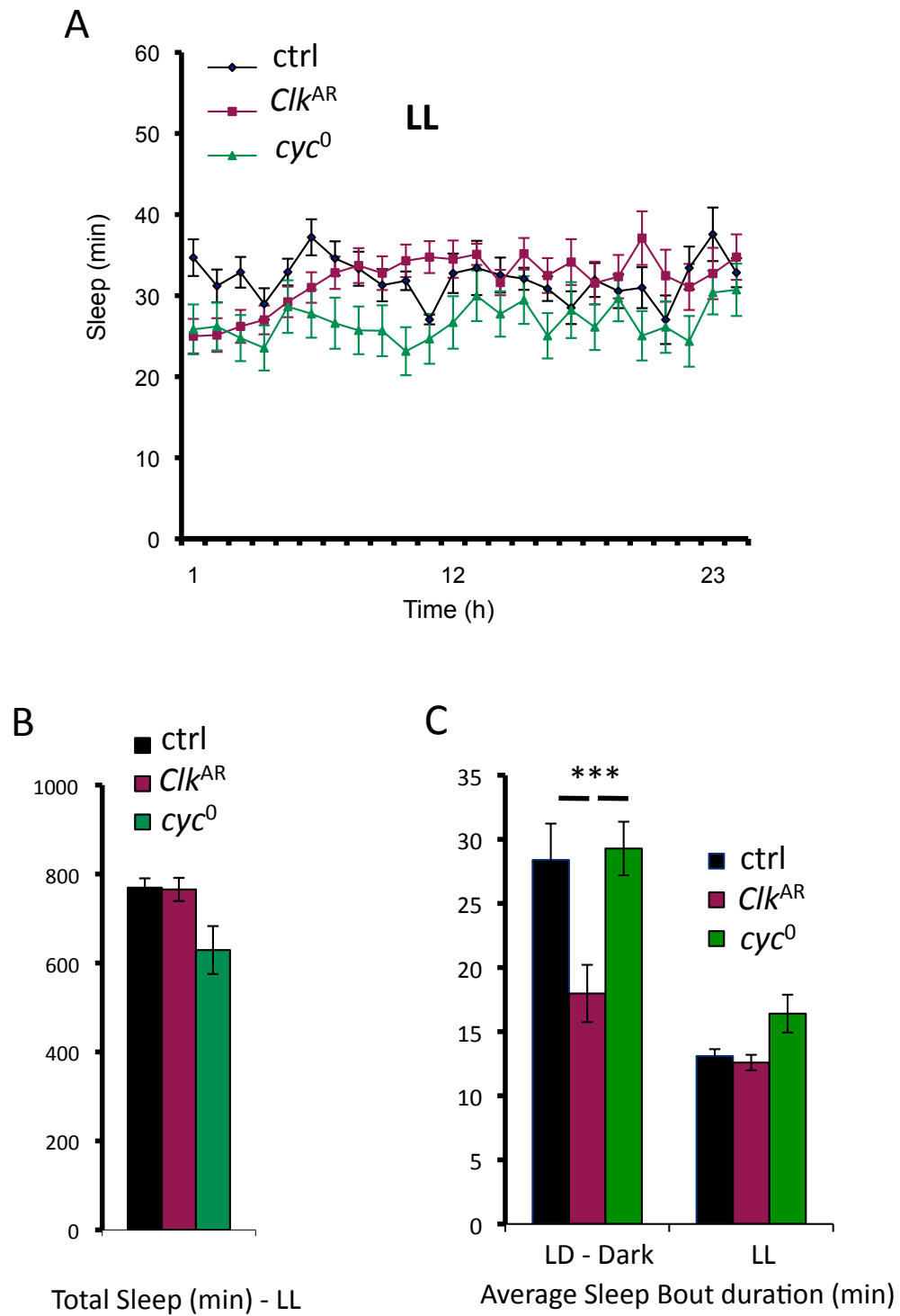

Figure S3

Supplement: S3 Fig — (A) In LL, both control, ClkAR and cyc0 flies lose sleep rhythms. Total sleep (B) and sleep bout duration (C) were not significantly different between genotypes. (C) Compared to LD, average sleep bout during the presumptive night was reduced for controls and cyc0 in LL. (PDF) [file pgen.1006507.s003.pdf]

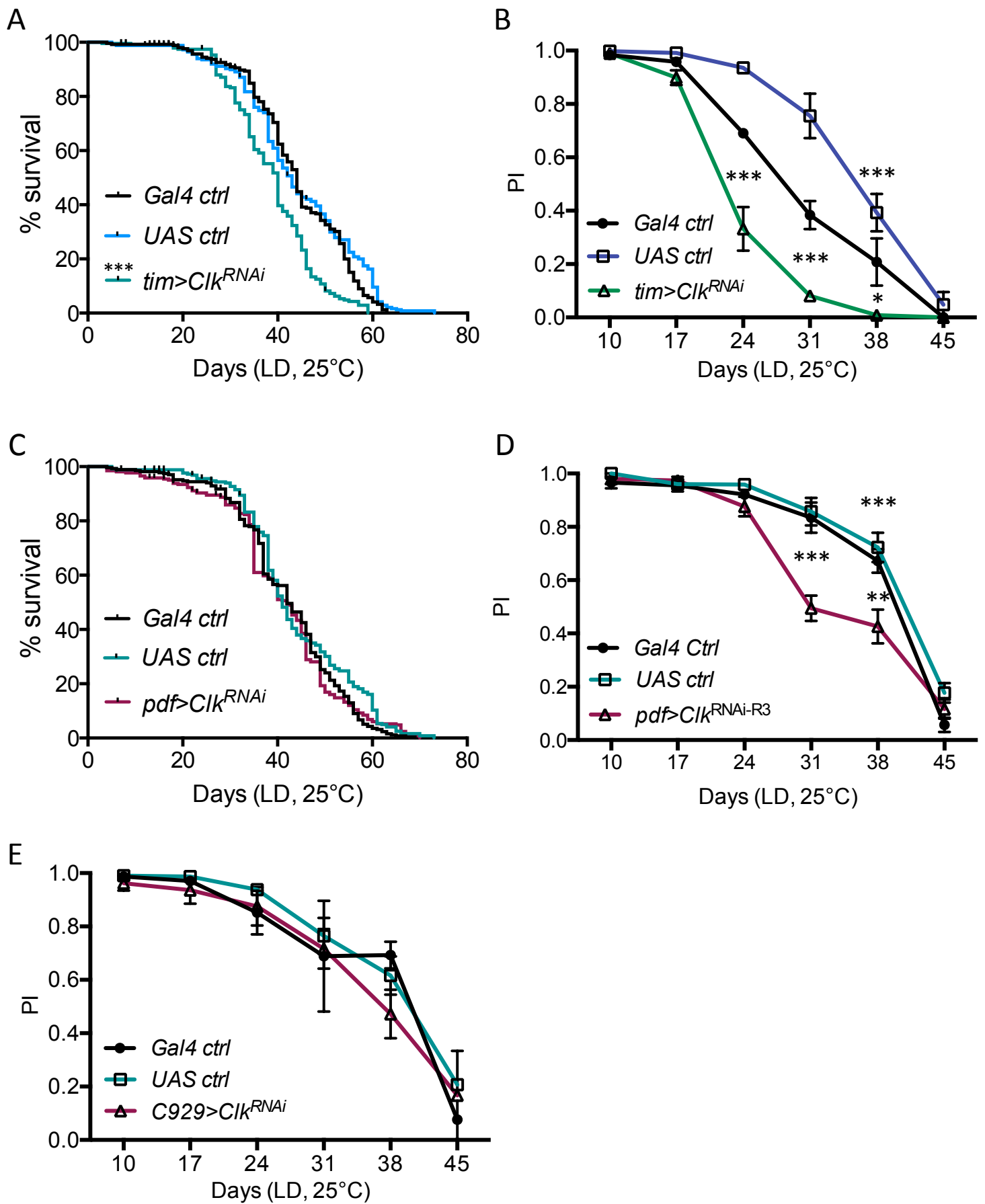

Figure S4

Supplement: S4 Fig — ClkRNAi expression in all clock cells (tim>ClkRNAi) decreases longevity (A) and strongly accelerates ARLI (B). Note that ARLI of the tim-Gal4/+ control is also impaired. (C) Expression of ClkRNAi in PDF neurons does not affect longevity. (D) Using a second ClkRNAi (UAS-ClkRNAi-R3) to inactivate Clk in PDF neurons also impairs locomotor performances. (E) Expressing ClkRNAi in the l-LNv (C929>ClkRNAi) had no consequence on SING decline. (PDF) [file pgen.1006507.s004.pdf]

A

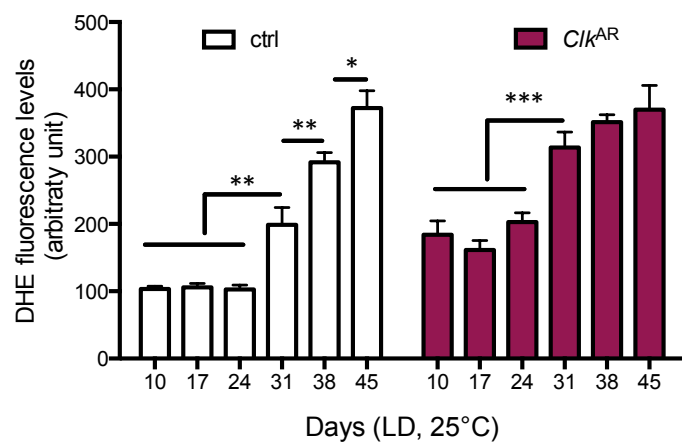

B

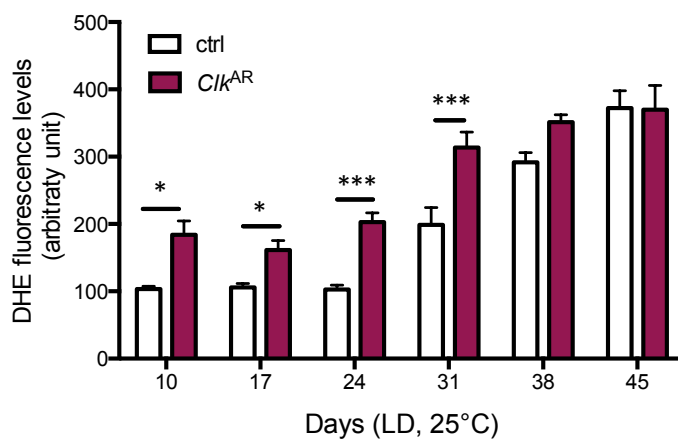

Figure S5

Supplement: S5 Fig — (A) Brain ROS increase with age between days 24 and 45 post-eclosion in control flies and between days 24 and 31 in ClkAR mutants. (B) ClkAR flies exhibit higher brain ROS than controls from day 10 post-eclosion to day 31. Both panels show the same data arranged differently to facilitate comparisons. (PDF) [file pgen.1006507.s005.pdf]

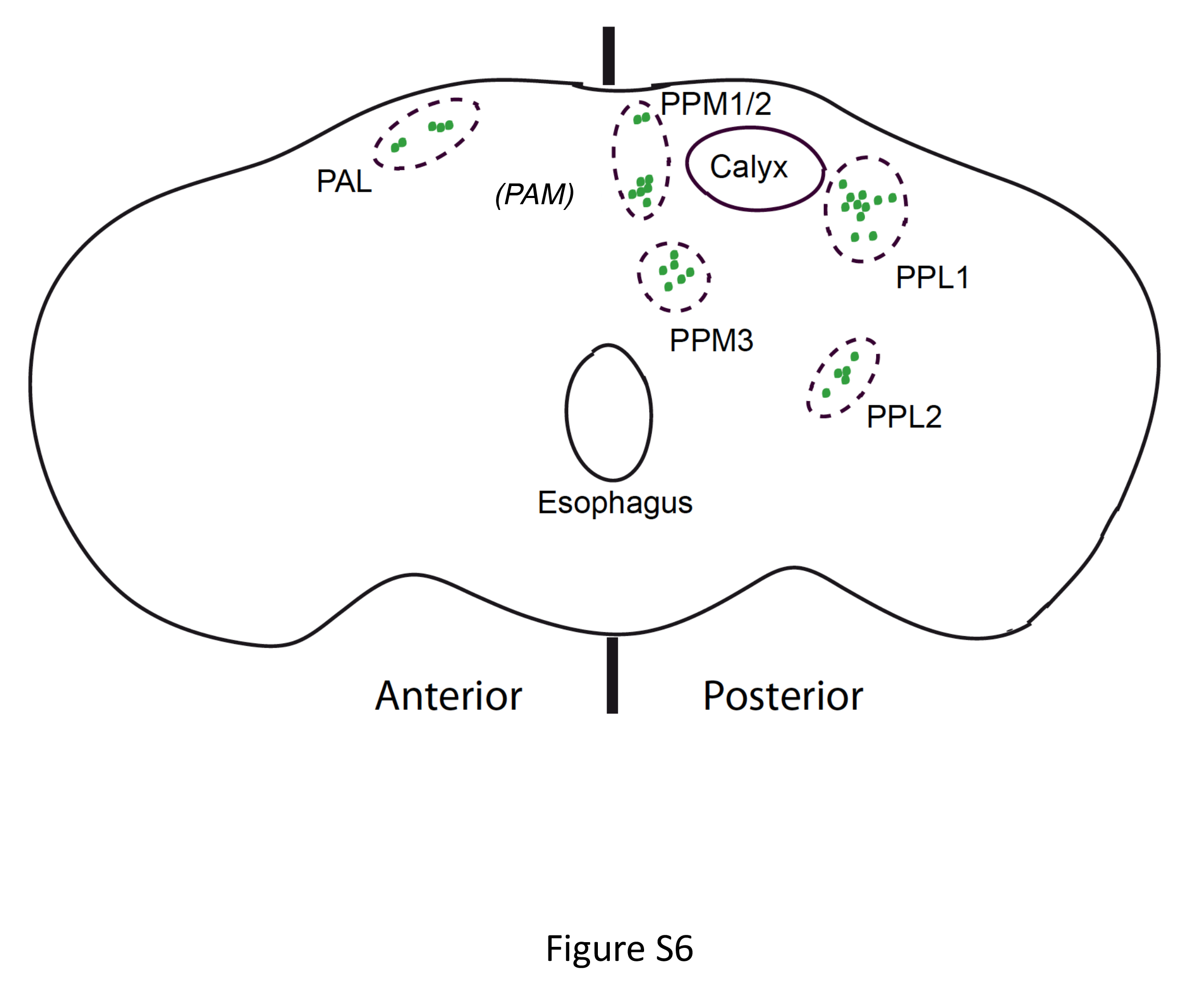

Supplement: S6 Fig — The hemisphere on the left corresponds to an anterior view, the right one to a posterior view. Approximate positions of esophagus and calyx of mushroom body are shown to provide orientation cues. On the left, only the approximate position of the large PAM (protocerebral anterior median) cluster is indicated, as we did not attempt to count its more than 100 neurons [36]. Other abbreviations: PAL, protocerebral anterior lateral; PPM1-3, protocerebral posterior median 1–3; PPL1-2, protocerebral posterior lateral 1–2. (TIF) [file pgen.1006507.s006.tif]

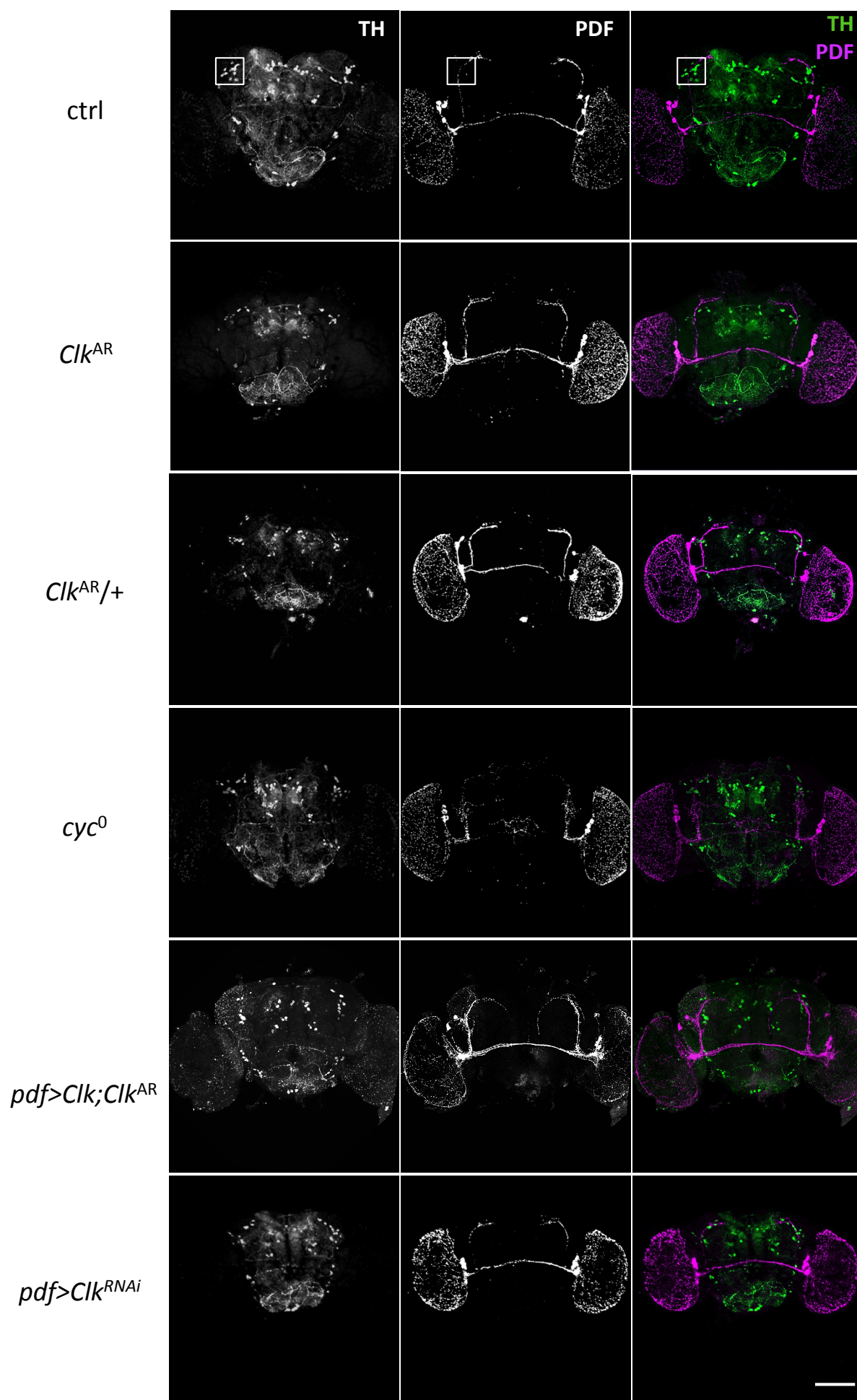

Figure S7

Supplement: S7 Fig — Co-immunostainings against TH (green) and PDF (magenta) of whole-mount adult brains of aged Canton-S flies (ctrl) (31 days post-eclosion). ClkAR, ClkAR/+ and pdf>ClkRNAi exhibit normal s-LNv dorsal projections (PDF, magenta) but reduced TH-positive cells (TH, green) in the PPL1 cluster (boxed region in ctrl brain, see also S7A and S7B Fig). s-LNv dorsal projections are altered in cyc0 mutants. Scale bar: 100μm. (PDF) [file pgen.1006507.s007.pdf]

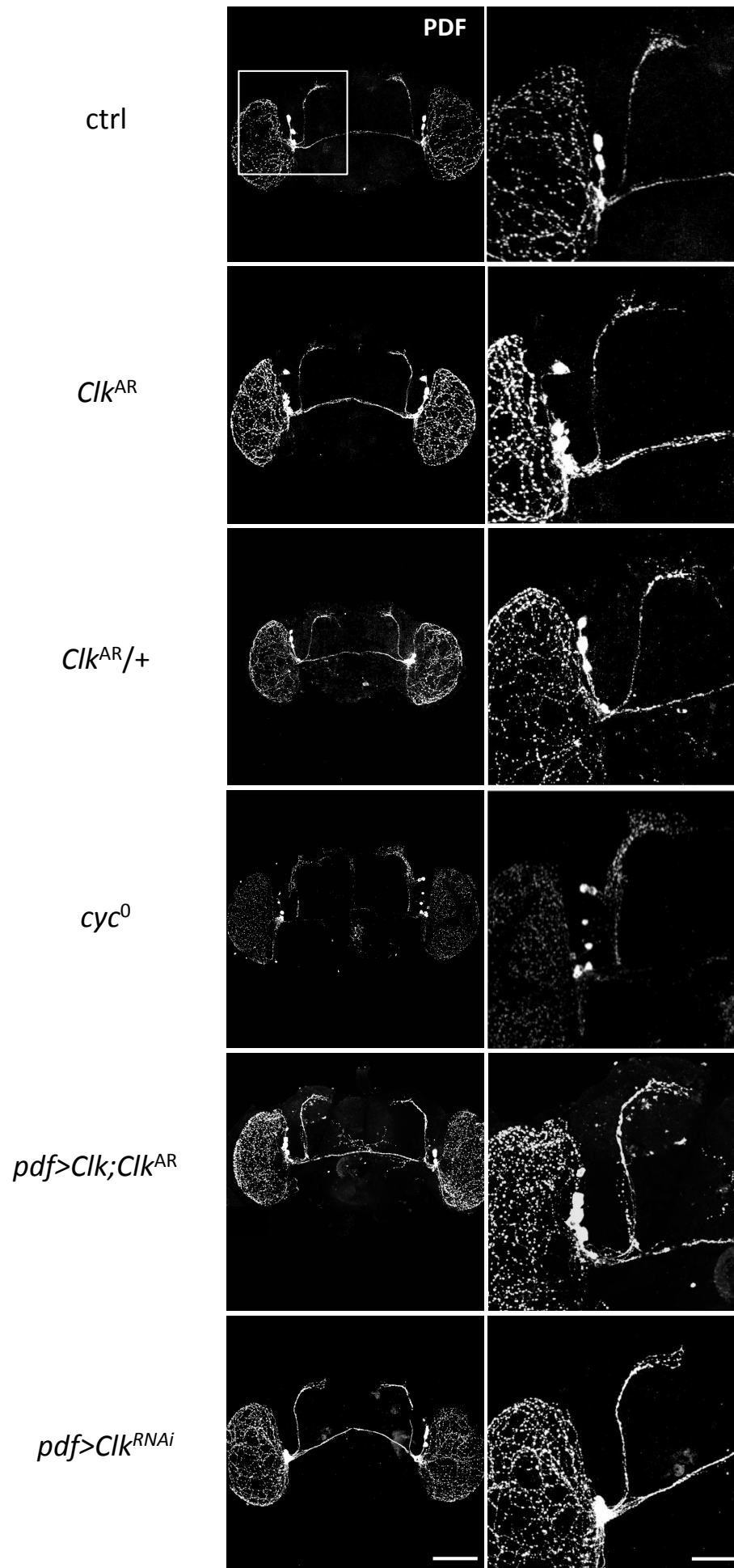

Figure S8

Supplement: S8 Fig — A different sample of the same genotypes shown in S7 Fig. They illustrate that s-LNv dorsal projections are reduced only in the cyc0 background. Scale bars: 100μm (left panels), 50μm (right panels). (PDF) [file pgen.1006507.s008.pdf]

A

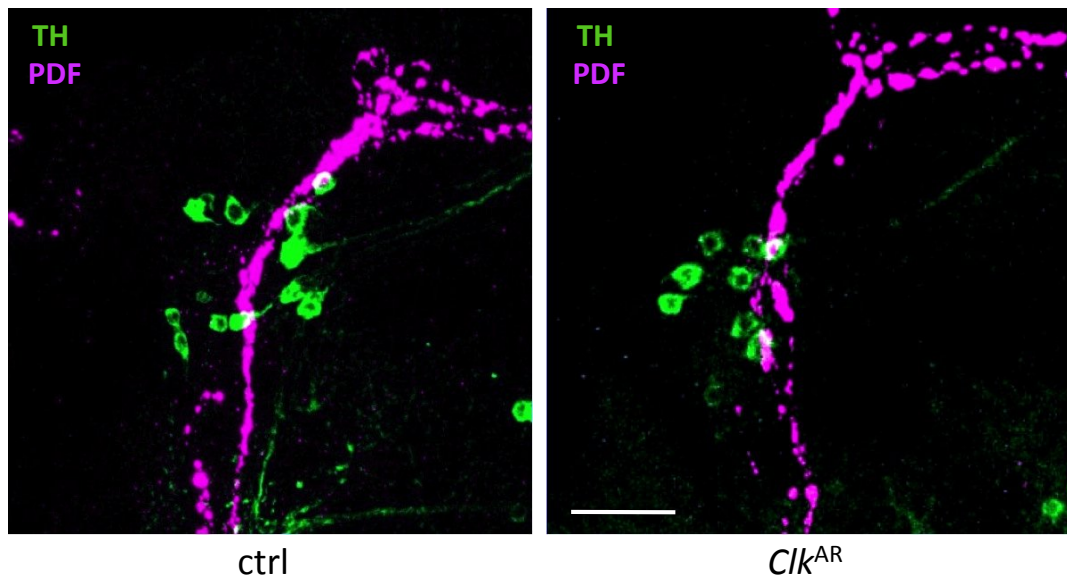

B

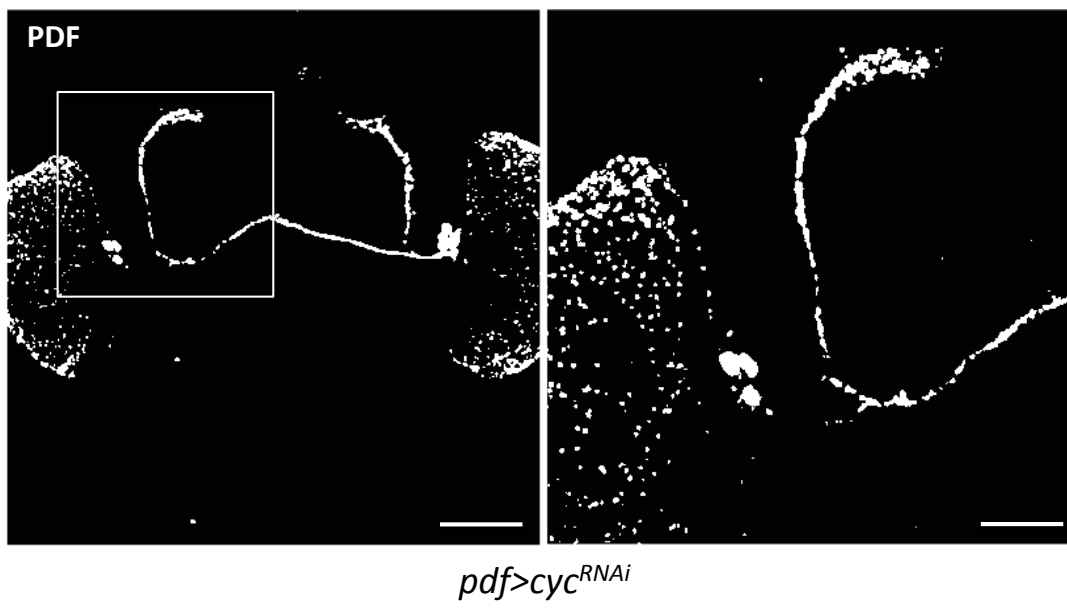

Figure S9

Supplement: S9 Fig — (A) Co-immunostainings against TH (green) and PDF (magenta) of whole-mount brains of 31-days-old flies at higher magnification, showing that PPL1 TH-positive cells (green) are localized in the vicinity of the s-LNv dorsal projection (magenta). The number of TH-positive cells is reduced in the ClkAR brain (right panel) as compared to the control brain (left panel). (B) cycRNAi expression in the PDF neurons does not disrupt s-LNv dorsal projections. Scale bars: (A) 25μm, (B) 100μm (left panel), 50μm (right panel). (PDF) [file pgen.1006507.s009.pdf]

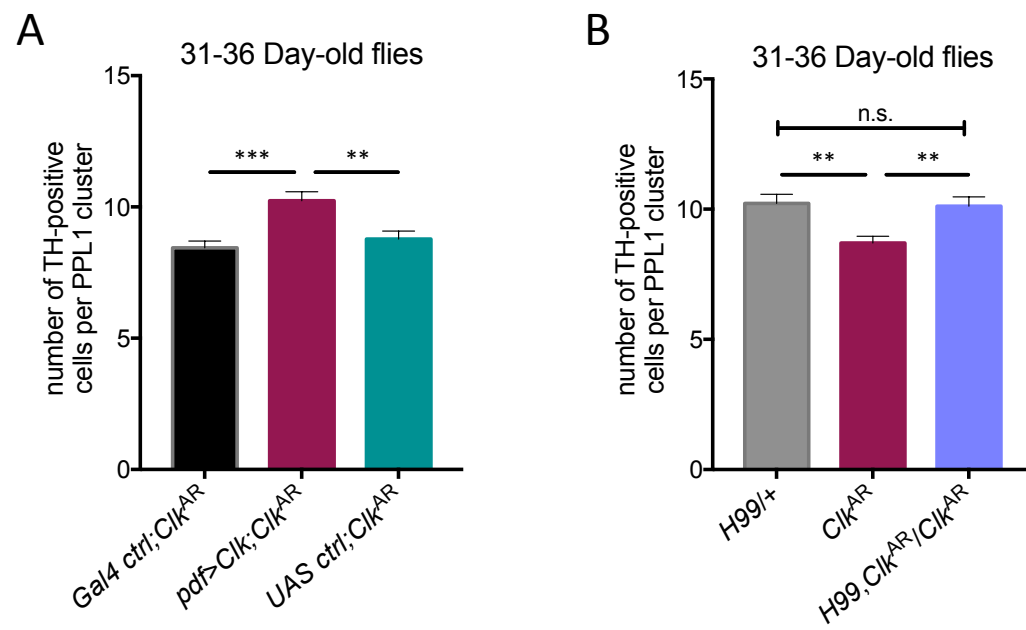

Figure S10

Supplement: S10 Fig — The number of PPL1 TH-IR neurons in the ClkAR mutant is rescued by restoring Clk expression in the PDF neurons (A), or by inhibiting apoptosis (B).TH-IR cells were counted in confocal stacks of brains dissected from flies of the indicated genotypes. Bars display the mean ± SEM of TH-IR PPL1 cell numbers, from 2 independent experiments, each with 8–10 brain hemispheres per genotype. The age of the dissected flies was 31 days in one experiment, and 35–36 days in the other. Although there may be a small effect of the H99 deficiency by itself, the ClkAR mutation has clearly no effect on the number of PPL1 neurons in the presence of that deficiency. (PDF) [file pgen.1006507.s010.pdf]

A

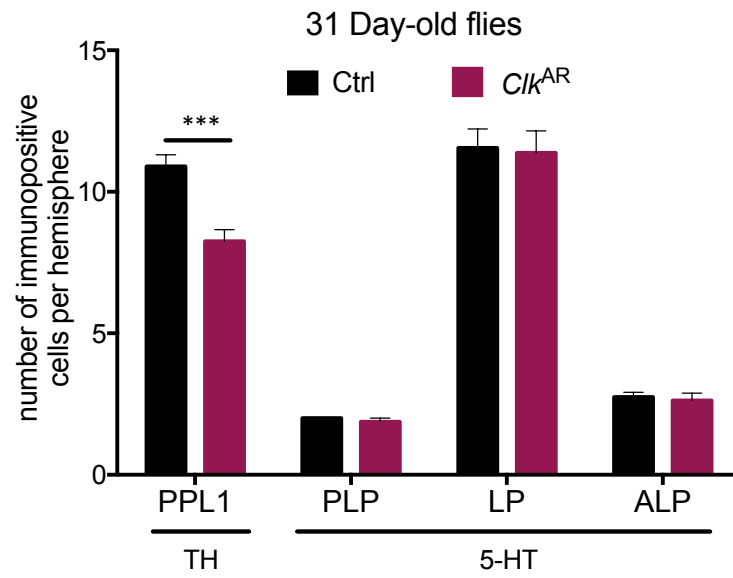

B

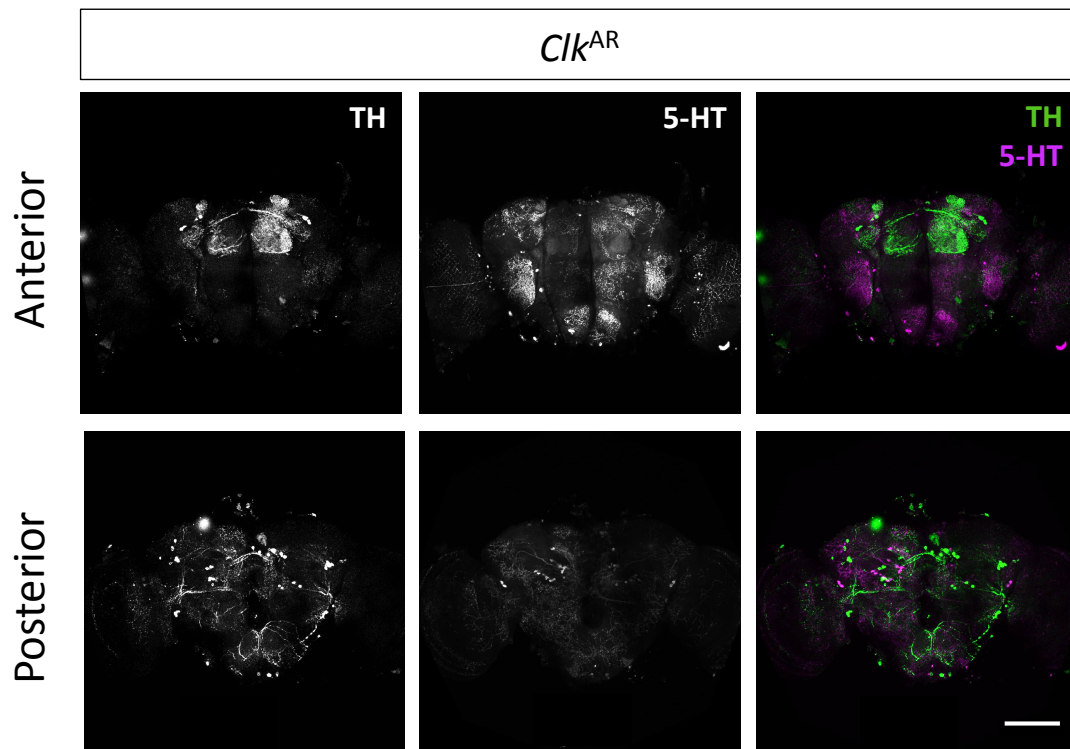

Figure S11

Supplement: S11 Fig — TH-IR and 5-HT-IR cells were counted in confocal stacks of brains dissected from 31-day-old control and ClkAR flies. (A) Bars display the mean ± SEM of TH-IR (PPL1) and 5-HT-IR (PLP, LP and ALP) cell numbers, from 8–10 brain hemispheres per genotype. ALP: Anterior Lateral Protocerebrum, LP: Lateral Protocerebrum, PLP: Posterior Lateral Protocerebrum [37]. (B) z-projections of the anterior and posterior parts of a representative 31-day-old ClkAR brain, double-labeled with anti-TH and anti-5-HT antibodies, as indicated. Scale bar: 100μm. (PDF) [file pgen.1006507.s011.pdf]
