# Supplementary material for: Drosophila Clock Is Required in Brain Pacemaker Neurons to Prevent Premature Locomotor Aging Independently of Its Circadian Function
Source: PLoS Genet. 2017 Jan 10;13(1):e1006507. doi: 10.1371/journal.pgen.1006507 (PMC5224980; doi:10.1371/journal.pgen.1006507)
Supplement: S1 Table — (DOCX) [file pgen.1006507.s012.docx]

| **Strains** | **p Value^a^** | **Median Lifespan**  **(Days)^b^** | **75th Percentile (Days) ^b^** | **Maximum Lifespan**  **(Days) ^b^** |
| --- | --- | --- | --- | --- |
| control |  | 43 ± 1.2 | 48 ± 1.8 | 67± 2.8 |
| *tim*^0^ | <0.0001 | 37 ± 2.0 | 42 ± 2.3 | 57 ± 2.0 |
| *cyc*^0^ | <0.0001 | 36 ± 1.5 | 44 ± 1.9 | 57 ± 2.1 |
|  |  |  |  |  |
| control |  | 45 ± 1.5 | 52 ± 1.7 | 66 ± 2.0 |
| *Clk*^AR^ | <0.0001 | 42 ± 1.6 | 45 ± 0.5 | 59 ± 2.4 |

**Table S1. Longevity of *Clk*^AR^ mutants (related to Fig. S1 A and B).**

^a^ p values were obtained with the log-rank (Mantel-Cox) test, on pooled data from 2-3 independent experiments.

^b^ Median lifespan, 75th percentile (the age at which 75% of the flies had died) and maximum lifespan were computed separately for 2-3 independent experiments. These 3 columns give the corresponding means and SEM.
