# Supplementary material for: Drosophila Clock Is Required in Brain Pacemaker Neurons to Prevent Premature Locomotor Aging Independently of Its Circadian Function
Source: PLoS Genet. 2017 Jan 10;13(1):e1006507. doi: 10.1371/journal.pgen.1006507 (PMC5224980; doi:10.1371/journal.pgen.1006507)
Supplement: S2 Table — (DOCX) [file pgen.1006507.s013.docx]

| **Genotype** | **n** | **%R^b^** | **Tau ± sem (h)^c^** | **Power ± sem^c^** |
| --- | --- | --- | --- | --- |
| Control (Canton-S) young^a^ | 20 | 100 | 24.3 ± 0.1 | 83.5 ± 6.1 |
| *Clk*^AR^ young | 20 | 0 |  |  |
| *cyc*^0^ young | 20 | 0 |  |  |
| *tim*^0^ young | 23 | 0 |  |  |
| Control (Canton-S) old | 16 | 88 | 24.9 ± 0.1 | 78.1 ± 5.1 |
| *Clk*^AR^ old | 24 | 0 |  |  |
| *cyc*^0^ old | 27 | 0 |  |  |
| *tim*^0^ old | 15 | 0 |  |  |
| *Clk*^AR^/+ | 21 | 100 | 24.5 ± 0.1 | 144 ± 4 |
| *cyc*^0^/+ | 29 | 100 | 24.2 ± 0.1 | 139 ± 4 |
| *han^5304^* | 12 | 50 | 22.8 ± 0.5 | 48 ± 11 |
| *han^5304^;;Clk*^AR^ | 31 | 13 | 24.8 ± 0.3 | 41 ± 9 |
|  |  |  |  |  |
| ***Gal4* controls** | | | | |
| *tim>w* | 32 | 100 | 24 ± 0.1 | 117.8 ± 2.9 |
| *pdf>w* | 21 | 100 | 24.9 ± 0.2 | 81.5 ± 4.1 |
| *C929>w* | 22 | 100 | 24.3 ± 0.1 | 100 ± 5.7 |
| *R6>w* | 24 | 82 | 23.4 ± 0.1 | 54.6 ± 4.9 |
| ***UAS* controls** | | | | |
| *w>Clk-B19* | 22 | 100 | 24.1 ± 0.1 | 125.1 ± 6.1 |
| *w>Clk*^RNAi^ | 21 | 100 | 24.2 ± 0.1 | 105.9 ± 5.8 |
| *w>Clk*^RNAi-R3^ | 34 | 100 | 24 ± 0.1 | 102.9 ± 4.4 |
| *w> cyc*^RNAi^ | 31 | 100 | 23.6 ± 0.1 | 83.2 ± 3.4 |
|  |  |  |  |  |
| *tim> Clk*^RNAi^ | 16 | 88 | 25.6 ± 0.2 | 102 ± 10 |
| *pdf>Clk-B19* | 23 | 93 | 23.6 ± 0.1 | 89.8 ± 8.9 |
| *pdf> Clk*^RNAi^ | 32 | 100 | 25.9 ± 0.1 | 158.2 ± 3.1 |
| *pdf> Clk*^RNAi-R3^ | 29 | 90 | 23.9 ± 0.2 | 72.9 ± 5.7 |
| *C929> Clk*^RNAi^ | 20 | 100 | 23.9 ± 0.2 | 84.1 ± 8 |
| *R6> Clk*^RNAi^ | 24 | 88 | 25.6 ± 0.2 | 45.1 ± 3.1 |
| *pdf> cyc*^RNAi^ | 32 | 8.7 | 17.3 ± 0.3 | 28.7 ± 0.7 |
| *tim> cyc*^RNAi^ | 31 | 6.5 | 25.5 ± 1 | 26.4 ± 4.5 |
|  |  |  |  |  |
| *pdf>w;Clk*^AR^ (*Gal4* control) | 24 | 0 |  |  |
| *w> Clk-B19*;*Clk*^AR^ (*UAS* control) | 23 | 0 |  |  |
| *pdf> Clk-B19*;*Clk*^AR^ | 24 | 18 | 23.3 ± 0.3 | 31.7 ± 4.9 |

**Table S2. Rest-activity rhythms in DD at 25°C.**

^a^ "Young" and "old" flies were placed into activity monitors 10 or 31 days post-eclosion, respectively. Locomotor activity rhythms were assessed over at least 5 days in constant darkness, which followed 5-6 days of entrainment in LD. When no age is mentioned, flies were usually placed into activity monitors less than 5 days post-eclosion.

^b^ Flies were deemed circadianly rhythmic if their period was 24h ± 4h, their power (height of the periodogram peak above the significance threshold) was > 20 and periodogram peak width was > 1.5h.

^c^ Periods and powers were obtained from the peaks in periodogram analyses. Only rhythmic flies, as defined above, were used to compute the mean period and power, and their sem, for a given genotype.
