# Supplementary material for: Drosophila Clock Is Required in Brain Pacemaker Neurons to Prevent Premature Locomotor Aging Independently of Its Circadian Function
Source: PLoS Genet. 2017 Jan 10;13(1):e1006507. doi: 10.1371/journal.pgen.1006507 (PMC5224980; doi:10.1371/journal.pgen.1006507)
Supplement: S3 Table — (DOCX) [file pgen.1006507.s014.docx]

| **Strains** | **p Value^a^** | **Median Lifespan**  **(Days)^b^** | **75th Percentile (Days) ^b^** | **Maximum Lifespan**  **(Days) ^b^** |
| --- | --- | --- | --- | --- |
| *tim>w* (*Gal4* control) | <0.0001 | 43 ± 0.7 | 53± 1.1 | 63 ± 0 |
| *w>Clk*^RNAi^ (*UAS* control) | <0.0001 | 44 ± 2.8 | 54 ± 2.1 | 73 ± 3.7 |
| *tim>Clk*^RNAi^ |  | 40 ± 2.1 | 45 ± 2.0 | 59 ± 0 |
|  |  |  |  |  |
| *pdf>w* (*Gal4* control) | n.s. 0.9253 | 42 ± 2.8 | 49 ± 1.8 | 69 ± 0.8 |
| *w>Clk*^RNAi^ (*UAS* control) | n.s. 0.0582 | 41 ± 1.1 | 52 ± 1.4 | 73 ± 0 |
| *pdf>Clk*^RNAi^ |  | 42 ± 2.8 | 48 ± 1.1 | 70 ± 0 |

**Table S3. Longevity of flies expressing *Clk*^RNAi^ in clock cells (related to Fig. S4 A,C).**

^a^ p values were computed using the log-rank (Mantel-cox) test between the corresponding control and the RNAi-expressing flies, on pooled data from 2-3 independent experiments.

^b^ Median lifespan, 75th percentile (the age at which 75% of the flies had died) and maximum lifespan were computed separately for 2-3 independent experiments. These 3 columns give the corresponding means and SEM.
